# The Multiscale Wisdom of the Body: Collective Intelligence as a Tractable Interface for Next‐Generation Biomedicine

**DOI:** 10.1002/bies.202400196

**Published:** 2024-12-02

**Authors:** Michael Levin

**Affiliations:** ^1^ Biology Department Allen Discovery Center at Tufts University Medford Massachusetts USA; ^2^ Wyss Institute for Biologically Inspired Engineering Harvard University Boston Massachusetts USA

**Keywords:** bioelectricity, biomedicine, collective intelligence

## Abstract

The dominant paradigm in biomedicine focuses on genetically‐specified components of cells and their biochemical dynamics, emphasizing bottom‐up emergence of complexity. Here, I explore the biomedical implications of a complementary emerging field: diverse intelligence. Using tools from behavioral science and multiscale neuroscience, we can study development, regenerative repair, and cancer suppression as behaviors of a collective intelligence of cells navigating the spaces of possible morphologies and transcriptional and physiological states. A focus on the competencies of living material—from molecular to organismal scales—reveals a new landscape for interventions. Such top‐down approaches take advantage of the memories and homeodynamic goal‐seeking behavior of cells and tissues, offering the same massive advantages in biomedicine and bioengineering that reprogrammable hardware has provided information technologies. The bioelectric networks that bind individual cells toward large‐scale anatomical goals are an especially tractable interface to organ‐level plasticity, and tools to modulate them already exist. This suggests a research program to understand and tame the software of life for therapeutic gain by understanding the many examples of basal cognition that operate throughout living bodies.

## Graphical abstract

Current biomedical interventions largely focus on bottom‐up techniques targeting the molecular hardware. Regenerative medicine will increasingly exploit top‐down approaches inspired by concepts in the behavioral and information sciences to target the collective intelligence of cells in vivo. The future of health sciences will involve collaboration and communication with the memory and decision‐making competencies of the agential material of life. This includes training cells and tissues for specific outcomes, biorobotics made of patient cells, and re‐setting anatomical outcomes via bioelectric interfaces.

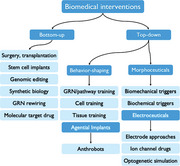

## 1 Introduction

While the current biomedical/pharmacological paradigm has become increasingly effective at controlling molecular‐level events with drugs, true regenerative medicine still eludes us, particularly in the area of anatomical control. We are unable to predict and repair most classes of birth defects or restore damaged or missing organs and appendages. We resort to toxic chemotherapies in the face of cancer [1]. We cannot build complex organs or new biobots to desired specifications and we remain powerless to stop senescence and the degradation of our functionality over time.

While existing physiological interventions are often effective, at least in the initial stages of application, they also have well‐known limitations: pharmacological therapeutics are plagued with differential utility across patients, diverse/unpredictable adverse events, and habituation. In addition, existing drug interventions mostly address symptoms, which often return or worsen once the drug is stopped, or even with continued treatment. The most successful current interventions target “invaders” in the body: microbes (antibiotics/antivirals and parasites (antiparasitics). Few treatments definitively and reliably induce permanent restoration of health by modulating the function of the host organism itself. How do we get to definitive repair?

I see the end‐game of this field as an “Anatomical Compiler.” Someday, we will be able to sit in front of a system and draw the plant, animal, replacement organ, or novel biobot that we want—at the level of functional anatomy. We will be able to specify its geometric structure, and the system will output a set of stimuli that can coax cells to build exactly that. It will also output a communications protocol manual, indicating what further stimuli will control its physiological functions and enable permanent restoration of health states; similar protocols will be usable to maintain and restore the health of native organs in situ. In this vision, the anatomical compiler is not a 3D printer or genomic editing system: it is a translator—a communication device that enables us to offload the complexity of producing the desired outcome onto the cells, communicating and collaborating with the living material by specifying target states for the native machinery, whose fundamental nature is to seek allostatic goals [2, 3, 4, 5]. The ability to communicate structural and functional goals to groups of cells could erase the burden of birth defects, traumatic injury, cancer, and degenerative disease.

Why do we not already have an anatomical compiler platform, or anything remotely like it? Individual successes have been achieved; but—despite the deluge of molecular information and omics data over the last few decades—we are extremely far from being able to exert full, rational control over form and function. This is the consequence of underlying limitations of the molecular biology paradigm, which has served as the exclusive basis for standard approaches in Western medicine [6, 7]. The following are a few examples among many that demonstrate how far we remain from understanding the control of complex anatomies.

Baby axolotls have forelegs, while early tadpoles do not. Despite having sequenced genomes for both species, we cannot predict whether frogolotls—chimeras made of frog and axolotl cells—would have legs or if those legs would be made of axolotl or frog cells or both. In fact, we cannot even predict the anatomy of a *non*‐chimeric species from its genome alone; we can only generate a rough prediction by comparing it to the genome of a species with known anatomy. While we study the developmental roles of specific genes, we are often surprised; for example, why do cells thrive without highly conserved cell cycle and genome integrity genes and pathways [8]?

Planaria provide another example of where the current paradigm is insufficient [9]. Due to accumulation of somatic mutations over 400 million years of asexual reproduction driven by fission and regeneration, planarian genomes are chaotic and their cells are mixoploid [10]. They also contain huge numbers of highly plastic and proliferative stem cells—a situation normally assumed to imply a high risk of cancer. Despite that, they are champions of regeneration, are cancer resistant, and apparently do not age. This is the polar opposite of the outcome that the current paradigm of “genome drives function” would predict for the animal with the messiest genome, full of undifferentiated cells in the adult stage. Examples like this emphasize how far we are from understanding the regulation of large‐scale properties, even as we drill down into better and better molecular details. This limitation will become increasingly stark as tools such as CRISPR move beyond single‐gene phenotypes and confront the problem of which genes to edit to get a desired complex anatomical or functional outcome.

Here, I argue that the above barriers can be overcome by augmenting today's focus on the hardware of life with a research program that seeks to exploit the inherent physiological software of cells and tissues. Numerous examples of non‐neural memory, problem‐solving capabilities, and collective decision‐making [11, 12] reveal that our bodies are constructed as an architecture in which each layer of organization navigates its own problem space [13]. I suggest that the tools of cybernetics, behavioral science, and neuroscience can be brought to bear on the deep problem of biological control to yield therapeutic and bioengineering solutions [14, 15] far beyond neurons and their control of conventional “behavior” of motile animals in 3D space. Specifically, I argue that the traditional modalities for engineering with passive matter are insufficient in the life sciences; living bodies are an agential substrate, full of competencies and agendas [16], and demand engineering approaches that are new to somatic biomedicine but have been used extensively and successfully in the behavioral sciences [17].

My position is pragmatic and naturalist (Box 1), with two main tenets: (1) we must understand and exploit the mechanisms appropriate to each level, and (2) empirical success in biomedicine and bioengineering should be the only arbiters of conceptual frameworks. At the same time, it is a deeply organicist position because the future of biomedicine and of basic biology requires us to come to grips with what is special about living materials and how they handle the scaling of intelligence across subsystems. Importantly, I am not arguing against the utility of the mainstream paradigm; rather, I hope to show how the focus on molecular events can be incorporated as one approach within a richer and wider set of powerful tools.

**BOX 1 | Beyond the mechanist/organicist dichotomy**

A fundamental argument has raged across the biosciences for centuries. The mechanistic approach sees living beings as a kind of machine, focusing on data about molecular components, research agendas that emphasize decomposition into parts, and emergence and complexity science as the key tools with which to predict and control systems [255, 256, 257, 258, 259]. In contrast, the organicist approach seeks proof [256, 260, 261, 262, 263, 264, 265, 266] that autopoietic living things are fundamentally different than machines, emphasizing top‐down causation and control, and unique features of life that cannot be captured by algorithmic models. Which framing is more conducive to the next generation of regenerative medicine? I argue that it is neither, as both take on unnecessary baggage that constrains future discovery by limiting the toolbox that workers in the life sciences can use.

**FIGURE 1:**
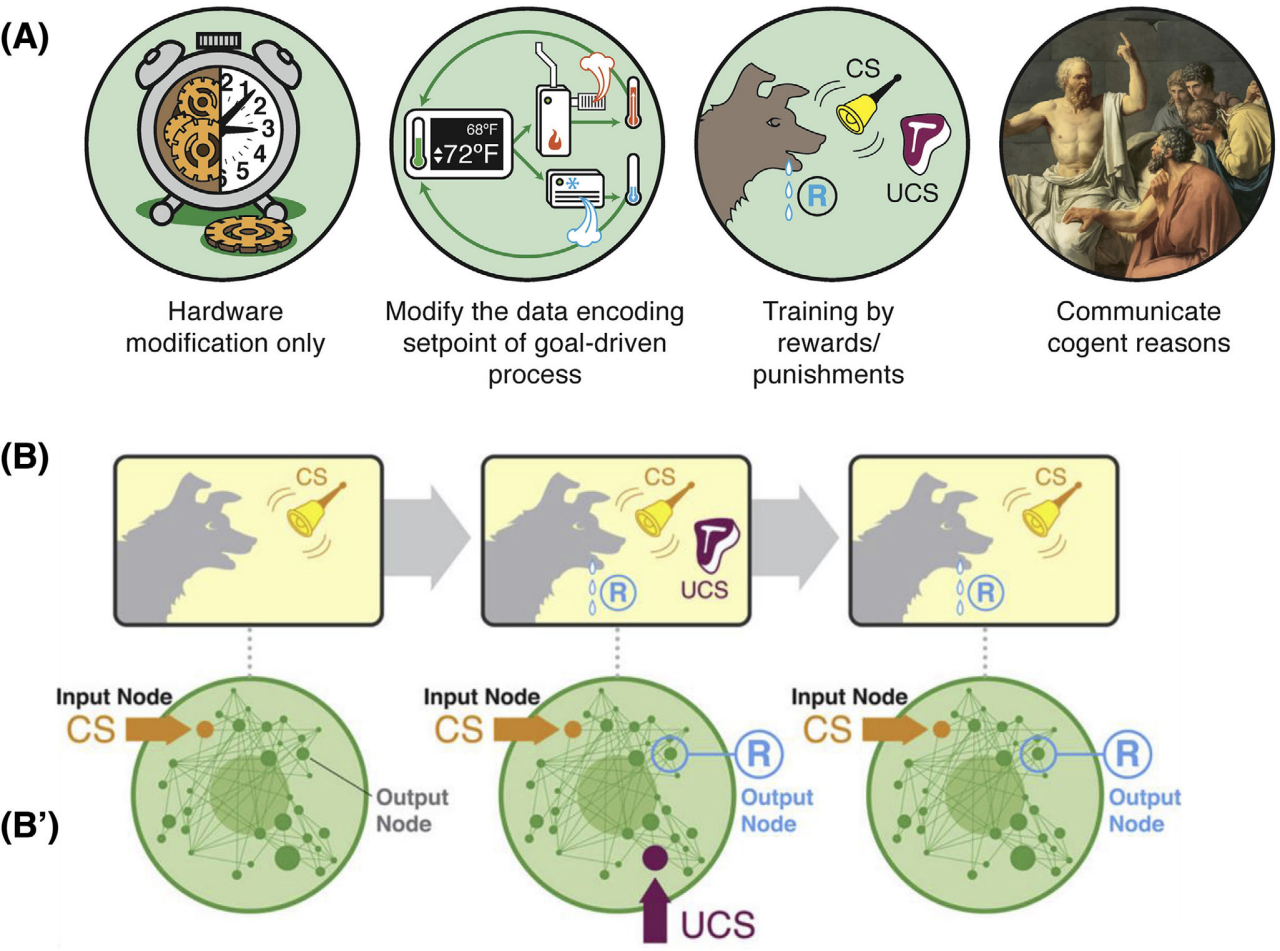
Living substrate on the spectrum of persuadability. (A) Systems fall on a “spectrum of persuadability,” which defines the kind of tools to which their prediction and control are amenable. Here are shown just four representative regions on that spectrum. Simple systems are only tractable to physical rewiring: changes of the hardware, which means that the engineer must understand every element and all of their emergent system‐level interactions. Homeostatic circuits enable simple goal states, and can be manipulated via the tools of cybernetics and control theory: rewriting their setpoints and letting the system achieve them. What is needed for these kinds of systems is knowledge of where and how the setpoints are stored and interpreted. More complex systems can learn, enabling the tools of behavioral science (e.g., behavior‐shaping) to achieve outcomes that are too complicated to be implemented directly. In these systems, the learning interface provides an abstraction layer that hides the underlying detail (which is managed by the learning algorithm), enabling the effective training of such systems without knowledge of the mechanisms under the hood. The far right end of the spectrum belongs to advanced symbol manipulating systems with complex meta‐cognitive circuits that can change their own goals, choose novel problems to solve, and otherwise exert their own self‐modifying agency to various degrees. Overall, moving rightward across the spectrum, the amount of micromanagement needed (or even possible) falls, moving from bottom‐up to top‐down control strategies, and the ability to depend on the system for autonomous problem‐solving rises. Crucially, what also rises is the importance of taking the perspective of the system itself (what does it know, what are its memories, and how does it make decisions) in order to have good predictive and control capabilities, while the need to understand every molecular component falls. Image courtesy of Jeremy Guay of Peregrine Creative previously published in [18]. (B) Crucially, the position of any given system (e.g., single cells) on this spectrum is not a philosophical question but an empirical one, settled only by making hypotheses and determining what level of control they afford. Here is shown an example [23, 24] in which the standard approaches of behavioral science (in this case, associative learning) are applied to what seem initially to surely be low‐agency, mechanical systems: gene‐regulatory networks (B’). They are deterministic, small networks of molecules that up‐ and down‐regulate each other's activity, and are currently addressed in biomedicine and synbio research exclusively via hardware rewiring, for example, gene therapy and promoter editing. But taking seriously the idea that unexpected proto‐cognitive capacities (not merely complexity) can be emergent in simple systems enables an empirical test in which such networks are exposed to patterns of stimuli one would use to train an animal, revealing several kinds of learning (which does not require changes to the hardware) including Pavlovian conditioning. Image courtesy of Jeremy Guay of Peregrine Creative, previously published in [24].

The perspective presented herein could be critiqued from both camps. On one hand, I argue to introduce strong forms of teleology (goal‐driven behavior) and cognitive capacities into molecular and cell biology as well as developmental and evolutionary biology. The use of tools from behavioral science to understand molecular pathways and morphogenesis is squarely against the mechanist project.On the other hand, I also liberally make use of computational tools (notions of software, reprogrammability, etc.) to understand life and uncover novel bioengineering capabilities, which is anathema to the organicist project that holds that such attempts miss what is special about life and mind. In prior work, I have suggested that “machines” and “living beings” are in fact on the same continuum (a ″spectrum of persuadability, Figure 1A), but that the cognitive aspects of this continuum reach way down, at least to the level of molecular networks. This view does not fit comfortably in either of the major camps.

I suggest two main concepts that dissolve the dichotomy between these positions and make them compatible in a way that facilitates future research [22]: pragmatism and pluralism. We should embrace the idea that everything in science (including such mechanist favorites as “pathways”) is a metaphor, and that our goal is the empirical test of which metaphors enable what outcomes. We cannot rely on philosophical commitments and stale categories that were developed in pre‐scientific times to constrain us from importing tools across disciplines. The mechanist/organicist debate [262, 263, 267, 268, 269, 270, 271, 272, 273, 274, 275, 276] rages largely because both camps believe they are describing things *as they are*. One especially divisive question is whether living things *are* computers, Turing Machines, and so forth. and thus demarcated by their known limitations. The knot is untied if we understand that cognitivist, computationalist, and other claims are about our *formal models, not about the system itself*. These are instead engineering protocol claims signaling the intention to use a particular framework to study the system. Nothing, especially living things, is objectively one thing—all we have are a multitude of approaches and metaphors, many of which can be appropriate (useful) depending on context. An orthopedic surgeon should see their patient as a mechanical machine. Their psychotherapist should not. Both are correct. As a key element of context, the notion of an “observer” is central; in biology, observers are scientists but also conspecifics, parasites seeking to hack a system, and the living subsystems of the body seeking to make sense of each other's signals [277].

I propose that it is critical to dissolve binary categories in favor of a continuum hypothesis with respect to cognition and a commitment to uncover the principles of scaling of competencies: not whether something is/is not cognitive, but how much and what kind of cognitive competencies it has that can offer a useful interface. The future lies in telling better stories about how cognition scales and about symmetries of deep concepts applied to novel substrates and levels of organization and size. In service of this goal, we need tools from both the mechanist and organicist toolboxes. Current computational paradigms do not capture everything that is important about life: self‐reference, self‐construction, blurring of the data/machine distinction, allegiance to on‐the‐fly salience of information, and much more. This means we must improve those paradigms for use with some kinds of systems. But other computational constructs—virtualization, abstraction layers, encryption, modularity, software, reprogrammability—are useful, and we should draw on them without fearing that using them commits us to the idea that they provide access to everything.Likewise, the utility of modeling an inner perspective with memories, goals, preferences, and decision‐making competencies is a perfectly rigorous approach to physical systems. The acid test of this framework, as with all mechanistic and organicist approaches individually, is empirical testing. Specifically, not whether each viewpoint can be fitted with epicycles to explain new biological discoveries post‐hoc, but whether it *generates* new research agendas and leads to the experimental discovery of new capabilities—whether it facilitates questions that could not be asked before.

While I freely make use of metaphors—perspectives on data that suggest next experiments—that have proven effective in areas such as computer and information sciences [18], I do not argue that living material is a computer in the sense that it uses anything like today's mainstream computer architectures, or that the typical Turing Machine model is sufficient to deal with self‐assembling, self‐modifying, multiscale goal‐driven living systems [19]. What I do claim is that our approaches to medical treatment have long been constrained by the implications of four connected, dominant ideas: (1) that the genome determines final phenotypic outcomes, (2) that intelligence only applies to brainy organisms and thus conceptual tools suitable for simple mechanisms at the level of chemistry are the only correct approach to the biomedicine of the body, (3) that goal‐centered frameworks are taboo because goals are the exclusive purview of advanced brains, and (4) that the control of biological form and function must occur via open‐loop emergence of complexity, arising bottom‐up from iterations of local mechanical rules.

The central hypothesis is that the body is a collective intelligence behaving in anatomical, physiological, and transcriptional spaces [13]. Thus, behavioral science tools developed to collaborate with complex systems should provide therapeutic advantages over biochemical micromanagement. Therefore, drugs and other interventions should be developed as communication messages, not low‐level controls. The focus for therapeutic discovery therefore must shift to understanding how proto‐cognitive systems interpret interventions, supporting a strategy of collaboration with endogenous capacities [20] to achieve desired outcomes.

Fortunately, we already have one well‐established modality for interfacing with complex goal‐driven systems: the bioelectric networks that tie neurons together toward conventional cognition also serve as a proto‐cognitive glue for somatic cells as they navigate anatomical spaces [21]. Powerful tools now emerging in other fields can be harnessed to communicate with these networks if we are willing to soften conceptual barriers that prevent the use of toolkits across categories. While it is very likely that other modalities (biochemical, biomechanical, and perhaps biophoton) also underlie similar dynamics in vivo, bioelectricity provides a uniquely tractable interface because of the advances of neuroscience in highlighting the ways in which electrophysiology can implement mind.

Here, I review concepts and data from the emergent field of diverse intelligence relevant to multiple scales in living organisms, frame morphogenesis and regeneration as behavioral outcomes of problem solving across these scales, and discuss how tools coming online in behavioral and systems neuroscience can revolutionize our understanding of molecular networks and cellular collectives as agential materials to enable transformative advances in definitive regenerative medicine, cancer, and senescence.

## 2 Intelligence Below the Cellular Level

I define intelligence here in William James’ [22] sense as the ability to reach the same goal by different means: the focus is on problem‐solving competency, which in this case is the ability to adapt to conditions to construct and repair a specific morphology or function. This definition of intelligence is functional and generic, enabling its study in substrates much different than we are used to. It is supported by an emerging body of work on basal cognition and diverse intelligence [11, 15], which finds applications of behavioral science tools to a wide range of systems along the evolutionary path from simple biochemical systems to the kinds of animals in which we recognize this capability in full bloom.

### 2.1 Conceptual Basis for Thinking About Tissue Intelligence

All systems can be categorized along a continuum corresponding to the degree of autonomy they implement (Figure 1A) [18]. It is often assumed that cells and tissues are at the very left of such a spectrum, as physico‐chemical machines which are complicated but low in agency. Accurate placement on this spectrum is critical because it determines the kinds of tools—conceptual and practical—that can effectively be used, and the kind of outcomes that can be expected. Neuro‐behavioral sciences illustrate how the tools for systems towards the higher end of the autonomy spectrum capitalize on inherent system capabilities: achieving a complex behavioral outcome in an animal does not require manipulating every neuron and muscle individually. Instead, we can simply train it because the system itself offers an interface—learning—which serves as an abstraction layer providing a convenient way to encapsulate complex, integrated responses behind simple triggers. Could something like this be possible for morphogenesis?

For bioengineers and workers in regenerative medicine to use this kind of approach to produce specific and complex anatomical forms and functions requires a crucial conceptual transition: the realization that living tissue is an *agential material*.

### 2.2 The Inner Life of Active Matter

A critical parameter for the continuum of agency is the degree to which an external observer must take into account the system's internal representation of the option space and its autonomous decision‐making. To know what a bowling ball is going to do on a bumpy landscape, your external, 3rd‐person view of the landscape, and calculations about energy minimization, tell the whole story. The same approach does not work for a mouse on that landscape—what is salient there is *its* view (internal model) of the landscape and free energy minimization with respect to its priors and goals, not yours [25, 26]. Where do cells fit on this continuum?

Single cells, from microbes to somatic cells, have been shown to have numerous proto‐cognitive capacities, including memory (learning), decision‐making, and anticipation [11, 27, 28, 29]. Especially relevant to future biomedicine are the examples showing that cells of multicellular organisms have not lost the basal features of adaptive information processing, including phenomena such as cardiac memory [30, 31] and active cell perception and signal processing with respect to stimuli [32, 33, 34, 35, 36, 37, 38, 39]. A diverse range of cell types exhibit learning via molecular signaling networks and bioelectric circuits [23, 24, 40, 41, 42] and context‐dependent decision‐making [36, 43, 44].

As will be seen below, the competencies of cell groups during morphogenesis arise from the scaling up of the single‐cell agentic repertoire. What underlies the competencies of cells? Does agential behavior first appear in single cells—are they the smallest unit of cognition [27, 45, 46, 47, 48]? It turns out that even below the single‐cell level, molecular components already have aspects that are tractable to the use of tools for the study of cognitive systems, and that what cells know (and what they can know)—their senome [45, 48, 49, 50]—is as important as their genomes, proteomes, and other such hardware specifications.

By applying standard approaches from behavioral theory [17], it was seen that even simple models of gene regulatory networks (GRNs) and molecular pathways show several different kinds of learning, including habituation, sensitization, and Pavlovian (associative) conditioning [23, 24]. By treating some nodes within the network/pathway as the unconditioned stimulus (UCS), other nodes as the response (R), repeated presentation of the UCS with an initially neutral node results in the network treating signals arriving on that node as a conditioned stimulus (CS) which can now trigger the response on its own (Figure 1B). This is a kind of dynamical state memory that does not require any hardware changes to the topology of the network or the strength of the edges (promoters). This is also a kind of “molecular placebo” because, due to its history of experiences, even a simple network can start responding to a neutral stimulus as if it was a much more potent one.

This shows how applying tools from other disciplines can reveal novel dynamics. The metaphor of gene networks as mechanical, low‐agency, dynamical systems [51, 52] is useful for many things but it did not on its own facilitate the discovery that GRNs and pathways should be trainable. Similarly, tools from cognitive neuroscience such as active inference are shedding light on reasons for the not‐infrequent failure of drug therapies [53, 54]. Importantly, for biomedical purposes, GRNs are an abstraction layer—an interface to cells that permits training them, just as brains and neural networks are an interface to animal behavior, one that was used by humans to train dogs and horses long before we knew how their brains worked. The implications of this molecular intelligence layer are numerous, spanning drug habituation, resistance of cells to therapeutics, unexpected side‐effects and differential efficacy across patients with different physiological histories, and the potential to use associative conditioning between drugs to induce outcomes using low‐cost and well‐tolerated trigger compounds [54]. The innate learning capacity of cells can lead to drug failure because the body's systems adjust to what they detect as a hacking attempt by an external exploiter. For this reason, first order models using conventional reagents to clamp a specific pathway state in place would not work—second order models, which take into account the cells’ ability to maintain goal states, and to facilitate re‐writing those goal states, are needed.

Given this learning capacity in chemical networks within cells, all the benefits of learning for evolution become relevant, modifying the speed and course of evolution beyond what standard cycles of random mutation + selection produce alone [55, 56, 57, 58]. Indeed, several computational studies have shown that evolution works quite differently over an agential material that has competencies and plasticity beyond a fixed genotype→phenotype relation [59, 60]. The more intelligent the software mapping, the more robust and rapid the evolutionary progress. Several studies have now identified ways in which exploratory learning and other problem‐solving capacities [40, 61, 62, 63, 64, 65] can affect evolution.

It is important to emphasize that we have only begun to scratch the surface of physiological problem‐solving. For example, when flatworms are exposed to barium—a nonspecific potassium channel blocker—their heads rapidly degenerate; this is not surprising given the necessity of endogenous potassium metabolism in the neurons and other cells of the head. Remarkably, they soon regenerate new heads which are completely adapted to the presence of barium [66]. Transcriptomic analysis reveals just a handful of genes that have been up‐ and down‐regulated to enable this feat of morphological and physiological homeostasis. The key question is: how do cells know *which genes to regulate to resolve their physiological stressor*? It is unlikely that they have a built‐in response to a frequent evolutionary history of barium exposure. As with the examples of polyploid newts and many other novel manipulations (Table 1, and the biobots described below), navigating a high‐dimensional transcriptional space to the right solution for barium exposure is an impressive problem‐solving capacity that we do not yet understand.

**TABLE 1:** Examples of morphogenetic problem‐solving competencies.

System	Competency	Reference
Frog metamorphosis	Highly abnormal tadpole faces (scrambled craniofacial organs) remodel to normal frog faces as structures move through novel paths and stop when the correct pattern is reached	[67]
Mammalian early embryos	Early embryos can be split, fused, or even injected with carcinoma cells, resulting in normal embryos	[68, 69, 70]
Frog, zebrafish, newt	Structures such as kidney tubules, nervous system, somites, and overall body reach normal shape and size despite induced changes in ploidy, cell size, and cell number	[71, 72, 73, 74]
Axolotl	Normal number of dorsal root ganglia form despite induced gains or losses of neural crest cells	[75]
Axolotl	Tails grafted to flank positions remodel into limbs, showing how local shape is governed by large‐scale bodyplan information adjusting to unexpected configurations as needed	[76, 77]
Cnidarian, simple chordate, and vertebrate embryos	Embryos find alternative developmental trajectories to a gastrulated embryo when the patterning and the topology of the embryo are altered	[78, 79, 80]
Mouse embryos	Growth and cell division systemically adjust to ensure catch‐up of long bone morphogenesis to compensate for induced cell cycle arrest	[81]
Vertebrate embryo limb development	Muscle bundles, tendons, and some nerves duplicate and adjust as needed to make functional fingers when ectopic bones are induced. Implanting a bead with growth factor that triggers cartilage condensation also results in ectopic joints and flexor and extensor tendons	[82, 83, 84, 85]
Mouse embryos	Genetic mutants with misrouted axons from the dorsal lateral geniculate nucleus still find their way to the visual cortex via alternate routes and reestablish a normal pattern of thalamocortical connectivity.	[86]
Goat embryo	Goat born without forelegs established many coordinated changes in pelvic structures needed for bipedal locomotion	[87]
Planarian regeneration	Planaria re‐established chemotactic sensing even after irradiation prevented creation of new cells after removal of auricles	[88]

Thus, cells have the capacity to solve problems in metabolic, physiological, and transcriptional spaces. But that is just the beginning of the impact that collective intelligence has on the basic and applied life sciences. Some of life's most remarkable and impactful capacities are revealed when we turn to the goals and competencies of large groups of cells, which traverse the latent space of anatomical possibilities.

## 3 The Collective Intelligence of Cell Groups

### 3.1 Morphogenesis as Behavior in Anatomical Space

**FIGURE 2:**
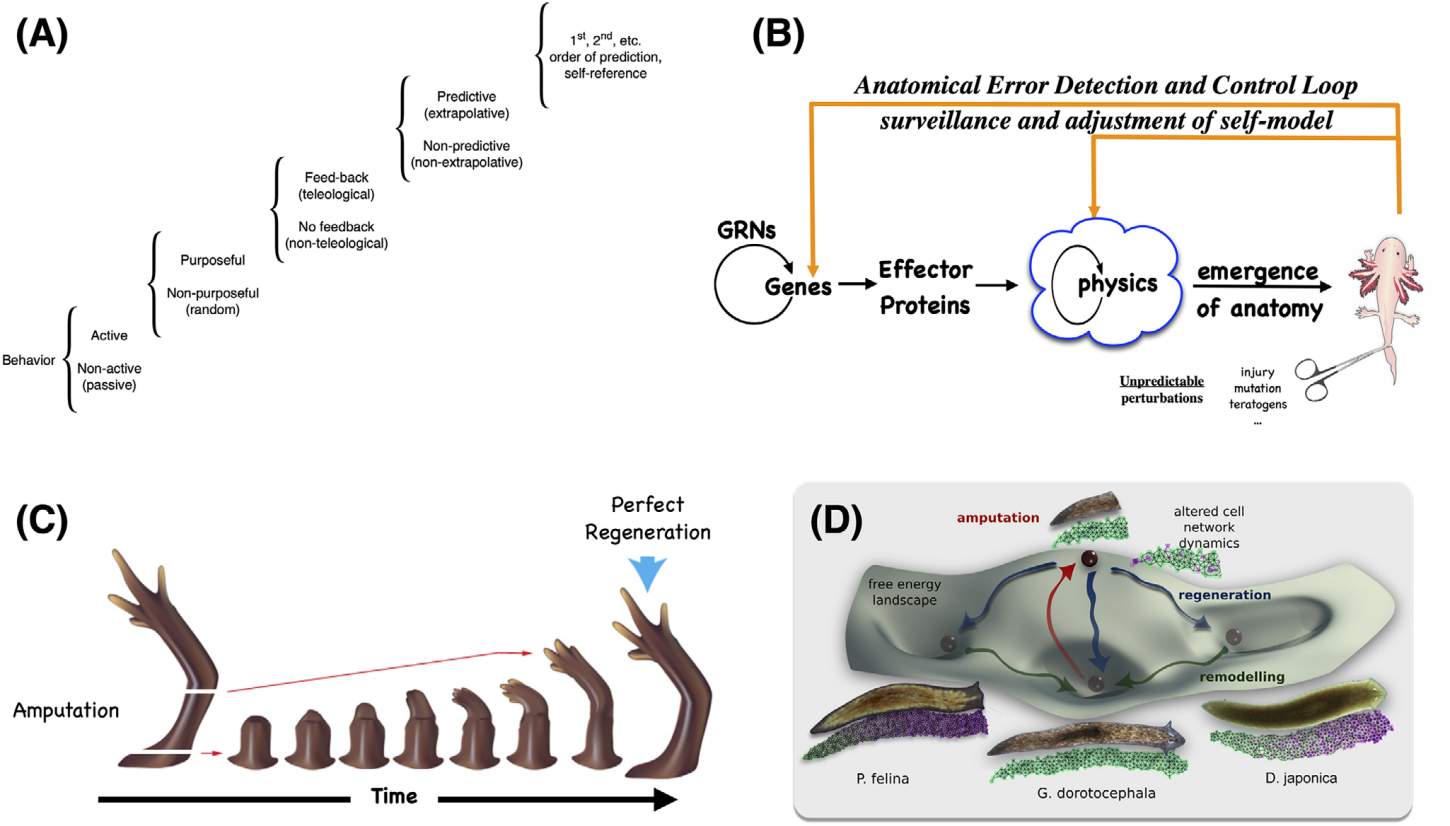
Unconventional intelligence. (A) Cognitive capacity lies on a spectrum [89] which spans from passive matter through diverse forms of active behavior with different degrees of causal linkage to past and future events. As one moves up the ladder, systems acquire more autonomy and require more consideration of their past and their internal model of the world (i.e., seeing the world from the system's perspective) to effectively control them. Image modified after [89]. (B) Anatomical outcomes are a combination of two fundamental processes. The more conventional and most often emphasized is feed‐forward (open‐loop) emergence of complexity: gene‐regulatory networks (GRNs) produce proteins which interact according to the laws of physics and eventually result in complex outcomes. However, a critically important and less often emphasized component is the ability of the system to activate effectors at the level of transcription and physiology to continuously reduce the error relative to the creature's target morphology. Image previously published in [90]. (C) An example of this anatomical homeostasis is seen in salamander limb regeneration, where an amputation anywhere along the axis causes cells to rapidly proliferate and undergo morphogenesis, stopping when (and only when) a “correct salamander limb” is complete. This closed‐loop process, which is context‐sensitive and most effectively described as a homeostatic loop with a specific setpoint, is fundamentally different than just emergence because it aims at a well‐defined, yet complex, goal state and specifically implies questions such as the mechanism of storage of the target morphology information and the degree of competency the system would have to reach its goal in the face of various different kinds of perturbations away from that setpoint. Image courtesy of Jeremy Guay of Peregrine Creative, previously published in [14]. (D) One powerful formalism for modeling the ability of systems to return to their setpoints, especially at higher levels along the hierarchy in (A), is through the concept of navigation of problem spaces. In this case, the anatomical morphospace is the latent space of possible shapes that can be built [91, 92]. For example, planarian species have different head shapes. Each specific head shape represents an attractor in morphospace, and cell collectives navigate the space to get to their correct species‐specific region if deviated by damage or undergoing embryogenesis. However, as with many kinds of autonomous navigational systems, the same hardware can occupy different attractors, and a genetically wild‐type planarian fragment can grow heads appropriate to different species of planaria [93, 94]. Image courtesy of Alexis Pietak, previously published in [94].

We previously argued that morphogenesis can be viewed as *behavior in anatomical morphospace* [13]; on this model, cellular collectives are systems that actively navigate from various starting states to a region corresponding to the species‐specific target morphology (Figure 2). As with all autonomous systems, the navigation process can exhibit diverse degrees of competency, ranging from a constrained random walk to highly advanced pathfinding and problem‐solving [89]. The practical benefit of entertaining this metaphor (in parallel to other equally metaphorical terms, such as “pathways” and “genes for traits”) is that it encourages a specific research program: the use of tools from behavioral and cognitive sciences that offer mature and powerful frameworks for understanding, predicting, controlling, and creating agents that navigate problem spaces.

From this viewpoint, neuroscience is not about neurons, but about understanding multiscale dynamics of emergent intelligence [95, 96]. If morphogenesis is decision‐making and goal‐directed behavior, then memories, preferences, measurements, disorders of perception, active inference, self‐models, and many other dynamics become potential therapeutic targets in development, regeneration, and cancer [15, 97].

### 3.2 The Competencies of Anatomical Homeostasis

Conventional intelligence results when brains exhibit problem‐solving by a collection of neurons bound together into a network which has memories, goals, and preferences far beyond those of the individual cells. In this sense, the brain is a collective intelligence too. The evolutionary precursor of this remarkable capacity is the ability of all cell networks, not just neurons, to solve problems by navigating anatomical morphospace in the progression from egg to adult. Perturbative experiments in this space, like those done to probe the intelligence of conventional behavior in 3D space, clearly indicate that this is not a hard‐wired process and reveal the ability of cell collectives to achieve their anatomical goals despite interventions that deviate them from their normal path.

A few instructive cases of morphogenetic problem‐solving are shown in Table 1. They have several fascinating aspects in common. First is the notion of anatomical homeostasis: the ability of systems to reach and maintain a specific region of anatomical morphospace despite deviations. These examples indicate that these processes are *not* entirely open‐loop: large‐scale feedback loops exist that measure distance from a given target state and execute diverse molecular steps to implement those goals (reduce error). If we can accept this evidence for the existence of setpoints and competency mechanisms, it opens the field up to the use of tools from cybernetics and control theory—sciences of physical systems with true goals. Second, they expand the well‐known examples of homeostasis in which the setpoint is a simple scalar (hunger level, blood pH, etc.), demonstrating that networks can store setpoints that serve as complex data structures (like rough morphogenetic specifications). A remarkable fact about homeostatic loops is that they implement valence (desirable outcomes) and preferences [98, 99]. Chemistry does not make mistakes—every chemical reaction is equally correct in following the rules of chemistry. But developmental biology, while consistent with the rules of chemistry underneath, brings in the notion of a birth *defect—*an outcome in which the system could not reach its target morphology despite efforts to do so.

A kind of creative problem solving enables life to handle novel scenarios by using the tools at cells’ disposal in new ways (a classic definition of intelligence). For example, “scrambled” tadpole faces remodel during metamorphosis to form a normal frog face, and polyploid newts made with multiple copies of the genome, and therefore larger cells, are of normal size because structures such as kidney tubules use fewer cells to make the same overall shape [71, 100]. Most remarkably, when the cells are made to be truly gigantic, a single cell may create the tubule, wrapping around and leaving the needed lumen in the middle. This can be analyzed as a kind of downward causation, in which under different conditions, distinct mechanisms (cell:cell communication and tubulogenesis vs. cytoskeletal bending) are triggered toward the same specific large‐scale outcome. It arises because evolution does not just make specific solutions to specific problems, it makes problem‐solving systems that do not over‐train on prior history, knowing that both environment and genetics will change over time [56, 101]. This enables incredible robustness and noise‐tolerance for development [56] and has fascinating implications for how we think about both evolution and applied biomedicine.

## 4 Bioelectricity as the Interface to Multicellularity

### 4.1 Bioelectric Networks: A Cognitive Glue

Conventional cognitive systems operate in problem spaces, and with goals, unknown to their parts. When a rat learns to press a lever to receive a reward, no individual cell had both the experience of interacting with the lever and that of getting the treat. The associative memory belongs to the collective, which is more than the sum of the millions of cells that comprise it. What underlies our memories, plans, and preferences is an electrochemical network that binds the functions of cells such as neurons into an emergent whole with novel properties. These networks comprise cells running electrical circuits determined by ion channel proteins (which set resting potential of each cell) and electrochemical synapses such as gap junctions (which determine how changes in those potentials propagate across the network).

One of the key properties of such networks is the storage of memory because voltage‐gated ion channels provide a kind of historicity in which transient physiological stimuli can induce long‐term changes in the bioelectrical property of the circuit [102]. Memories are ideal keepers of the setpoint information used by homeostatic behavior. Another key feature is top‐down control, which enables stimuli to kickstart complex downstream autonomous cascades. The most widespread application in bioelectricity—the cardiac defibrillator—works because it is possible to provide a stimulus and depend on the organ to take it from there. In terms of behavior, it is essential that simple stimuli can trigger multistep behavioral responses, including autonomous homeostatic loops that perform actions until specific conditions are met.

The final crucial thing about bioelectric networks is that they allow information to cross levels of organization and be remapped across problem spaces, for example between linguistic space and 3D motion space in active behavior. A human being's top‐level career and interpersonal goals are effectively pursued because those goals result in the movement of ions across muscle membranes that enable the organism to move. This ability of mental structures to control biochemistry is not limited to rare, exotic forms of biofeedback and placebo effects—such “mind‐body medicine” is the everyday miracle of voluntary motion, made possible by the bioelectric network that transduces high‐level mental patterns into the action of muscles and glands.

### 4.2 Thinking Beyond the Brain

While it is tempting to think of the cross‐level transduction as a unique capability of neural hardware and the electrochemical software that it enables, this architecture is ancient, being present in microbes [103]. Evolution discovered the immense benefits of electric networks by the time of bacterial biofilms [104, 105], using them to integrate physiological information across space and time in a colony. What did pre‐neural electrical networks think about before nerves and muscles evolved, enabling conventional behavior? Networks that managed an organism's position in 3D space evolved from non‐neural somatic precursors whose job was managing the navigation of the organism through anatomical morphospace [13]. Just as bioelectricity in the CNS binds neurons into a collective intelligence for motile behavior, somatic bioelectricity functioning from the time of fertilization binds all cells into a collective intelligence that solves morphogenetic problems.

**FIGURE 3:**
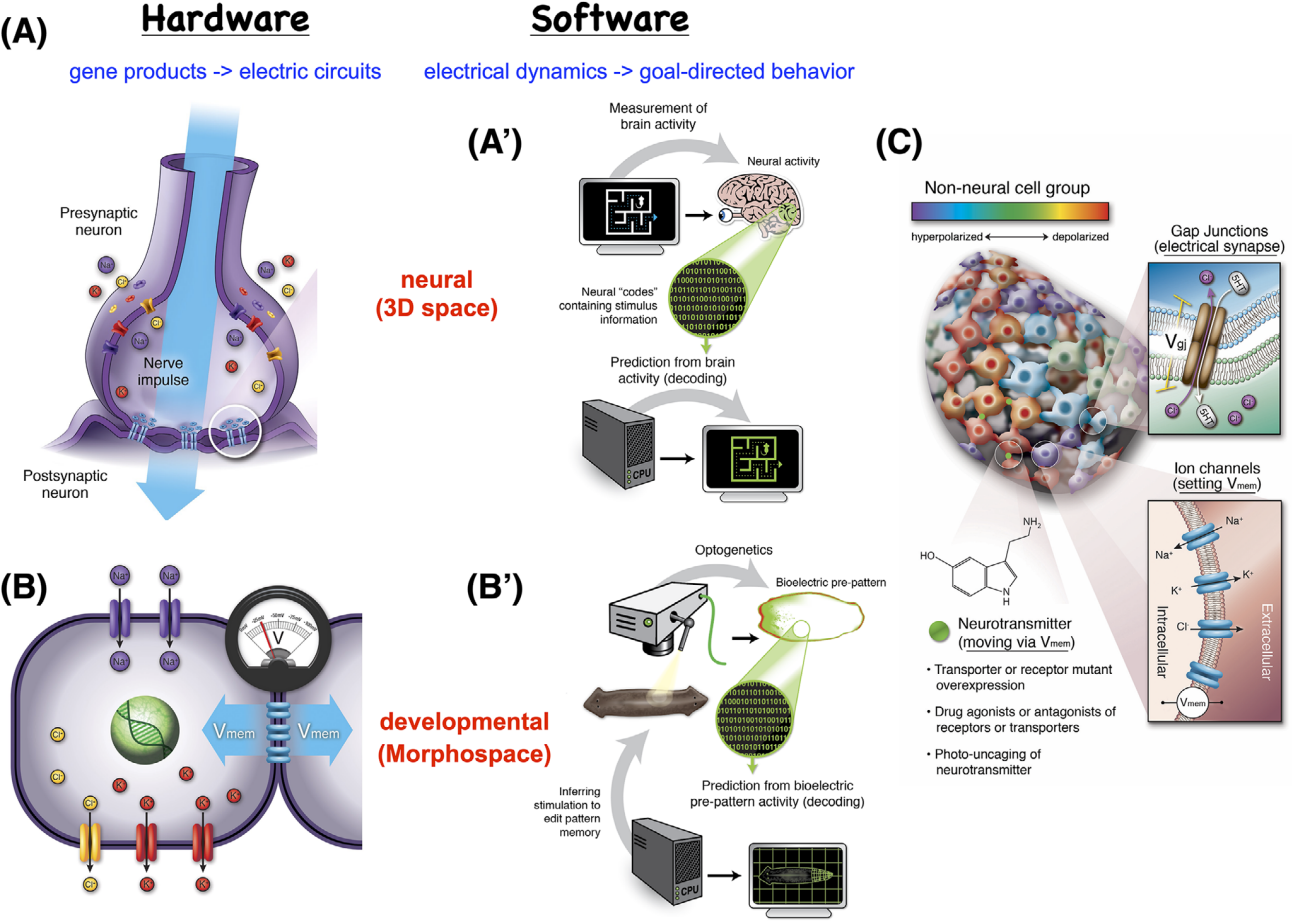
Developmental bioelectricity: fundamental mechanisms. (A) Neurons form networks that can perform computations and guide behavior in 3D space because context‐sensitive ion channels enable each cell to set and modify a resting potential (voltage gradient across the membrane) which it may communicate to neighbors if the (also context‐sensitive) electric synapses—gap junctions—are sufficiently open. This enables the project of “neural decoding” (A’) in which the physiological conditions are read out and decoded to read out the memories, preferences, and other aspects of the internal cognitive states of the being implemented by the neural network. (B) This machinery is evolutionarily ancient, with all body cells having ion channels and most having gap junction connections to a tissue network. Thus, the parallel task is cracking the bioelectric code to decode the proto‐cognitive content of the cellular collective intelligence, which uses precisely the same bioelectric and neurotransmitter mechanisms to navigate through anatomical space and solve problems (deal with injury and other perturbations). (C) Tools have now been developed to read and write the bioelectric state of non‐neural tissues, using molecular‐genetics, pharmacology, and light (optogenetics) to open and close ion channels and gap junctions, thus regulating the topology of connections in the network and the specific bioelectrical patterns encoded in it at any specific time. All images courtesy of Jeremy Guay of Peregrine Creative; A,B previously published in [90]; C previously published in [15].

Thus, brains and CNS function are the result of a fascinating evolutionary pivot. What changed was the space these networks represent and manage, and the time scale at which they operate (from the hours and days of morphogenetic change to the milliseconds of motile behavior). What stayed constant was the molecular machinery: ion channel proteins, electrical synapses (connexins), and neurotransmitter downstream targets that eventually regulate gene expression (Figure 3). Also, many of the algorithms (such as active inference [97, 106], perceptual multistability [107], dynamic rewritable memory [108], etc.) are highly conserved between morphogenetic and cognitive functions, enabling tools of computational cognitive science to be used in developmental biology contexts [15, 96].

Thus, the symmetry between developmental biology and neuroscience is deep [96, 109]. It can be seen in numerous channelopathies that result in patterning defects (see Table 1 in [110], and [111, 112, 113]), and in the applicability of the tools of neuroscience—from drugs to optogenetics to training protocols—to morphogenetic decisions.

### 4.3 Applications: Modulating Endogenous Bioelectric Cues

It has been clear for over a century that endogenous bioelectric phenomena play a functional role in the control of dynamic anatomical outcomes [114, 115]. But the development of molecular tools to read and write bioelectric state information in non‐neural tissues has led to the identification of the native genetics underlying the circuit properties, the molecular targets of the voltage change, and the processes that exploit bioelectric networks as a self‐modifying control network [116]. Focusing on spatiotemporal patterns of resting potential (complementing older work on electric fields and ion fluxes) revealed several types of effects. First is the control of stem cell differentiation decisions [117]. But the importance of bioelectricity really shines at the organ scale because the bioelectric code mapping patterns into anatomical outcomes is not a just cell‐level code.

**FIGURE 4:**
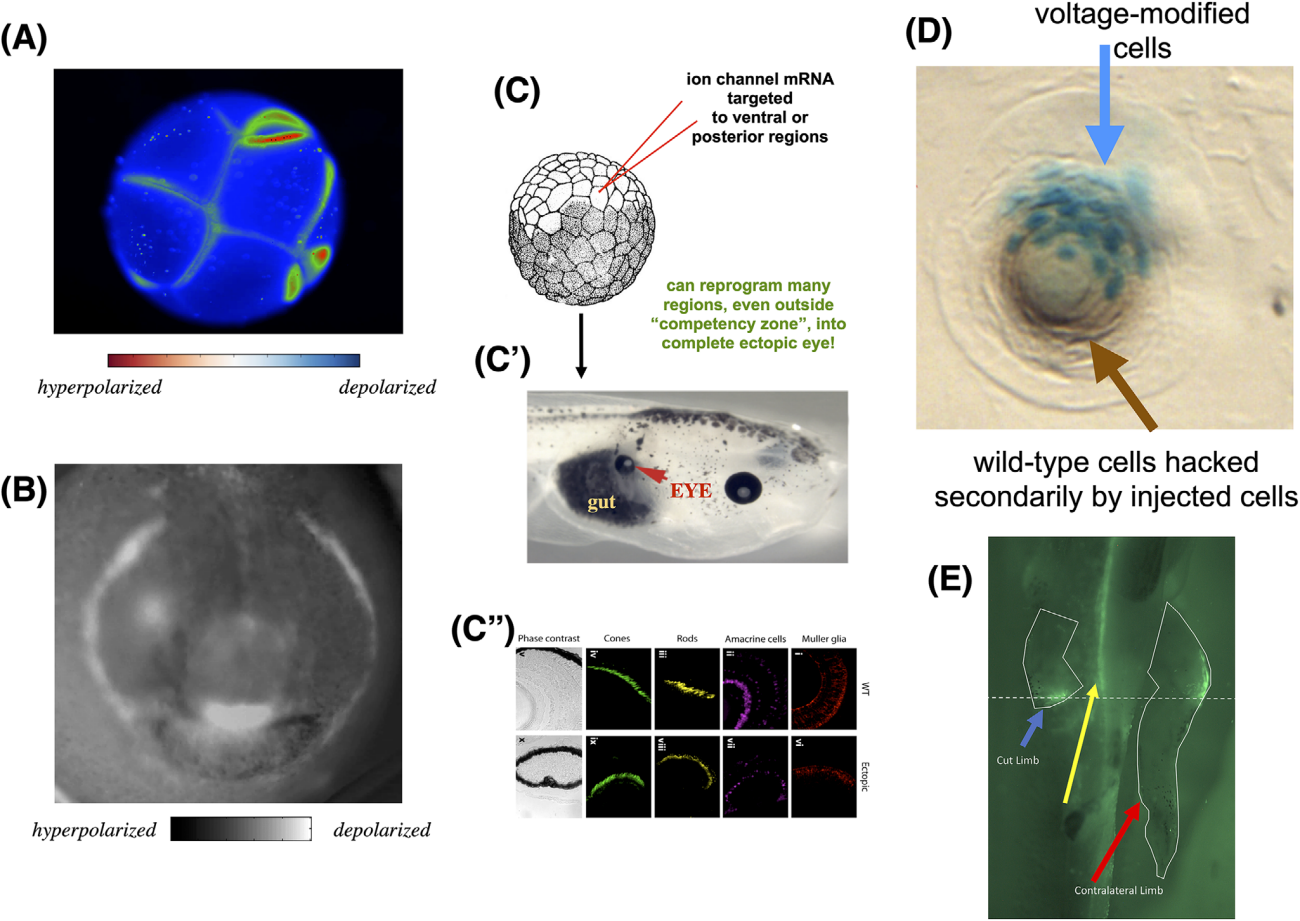
Anatomical outcomes enabled by reading and writing bioelectric prepatterns. Voltage‐sensitive fluorescent dye can be used to non‐invasively reveal the bioelectric patterns during morphogenesis. (A) Frog embryo cells during early development (image courtesy of Dany S. Adams). (B) The “electric face” prepattern which reveals the location of the eyes, mouth, and other structures that determine the locations of the gene expression domains that regionalize the craniofacial ectoderm and ultimately determine the position and size of the organs (previously published in [118]). (C) These patterns are functionally instructive, which is revealed by experiments such as injecting mRNA encoding potassium channels to induce a voltage spot resembling the eye field (left spot in B) in ectopic locations. This induces eyes to be formed (red arrow, C′) in ectopic locations such as in the gut. These eyes have the normal structure of internal components as seen in histological section and immunostaining (C″). Image in C taken from Xenbase [121]. Images in C′,C″ previously published in [120]. (D) Remarkably, when only a small number of cells are injected (blue beta‐galactosidase stain reveals the cells misexpressing the K^+^ channel protein), the task of making a lens in an ectopic location is still completed because these cells autonomously recruit neighbors (brown tissue) to complete the task for which the original cells’ numbers are insufficient. Image previously published in [18]. (E) A froglet soaked in voltage‐sensitive fluorescent dye reveals a depolarization signal (green stain above the dashed white line) in the un‐touched, contralateral leg (right arrow) which indicates whether, and where, the left leg was amputated (blue arrow). Yellow arrow indicates the spinal cord. Image previously published in [122].

At the organ level, bioelectric patterns serve as critical prepatterns—informational scaffolds that store rough anatomical setpoints for tissue‐level order [90]. One example is the electric face (Figure 4B)—an endogenous distribution of resting potentials that determines the gene expression and anatomical regionalization of the vertebrate face [118]. Manipulation of this pattern via pharmacology, optogenetics, or ion channel misexpression results in predictable changes to craniofacial development and explains why ion channel mutations lead to such phenotypes in models ranging from frog to human [119]. Recreating specific bioelectric patterns in new locations can also induce ectopic organs; misexpression of potassium channels in Xenopus laevis results in the formation of ectopic eyes [120] by recapitulating the voltage eye spot seen in the electric face (Figure 4C,D). This underscores the fact that bioelectric patterns are both *instructive* and highly *modular*—a simple signal induces a cascade of events to build a complex organ. It also reveals an interesting competency of the cellular medium: if too few cells are injected with the channel mRNA to build an eye (Figure 4E), they recruit neighboring cells. We did not have to engineer this ability, the material already does this, challenging us to exploit such capabilities and discover new ones.

A key point revealed by these data was that numerous regions in the posterior of the animal could be induced to form eyes, for example, on the gut. It was always thought that cells outside the anterior neural field in vertebrates were not competent to build eyes. But prior studies prompted cells with the so‐called master eye gene Pax6 [123]. It turns out that a higher‐level signal (*V*_mem_ change) can induce eyes almost anywhere in the body. Thus, higher‐level prompts can reveal new capabilities not apparent from manipulation of molecular levels.

The dynamic aspects of this organ‐level reprogramming have additional biomedical implications. At early stages in embryos injected with the potassium channel, many ectopic eye spots are detected, but just one new eye tends to appear at the end. This is the consequence of conversation between morphogenetic agents with different goals. The channel‐induced cells have been pushed toward a morphological setpoint corresponding to eye development, and try to convince neighbors to assist, via signals yet to be identified. Neighboring *uninjected* cells, whose morphogenetic setpoint is still “gut,” are sending signals to maintain the normal fate. This bidirectional communication to implement contradictory, physiologically specified goals provides important biomedical targets for challenges including birth defect repair, limb regeneration, and cancer suppression.

### 4.4 Birth Defect Repair and Limb Regeneration

**FIGURE 5:**
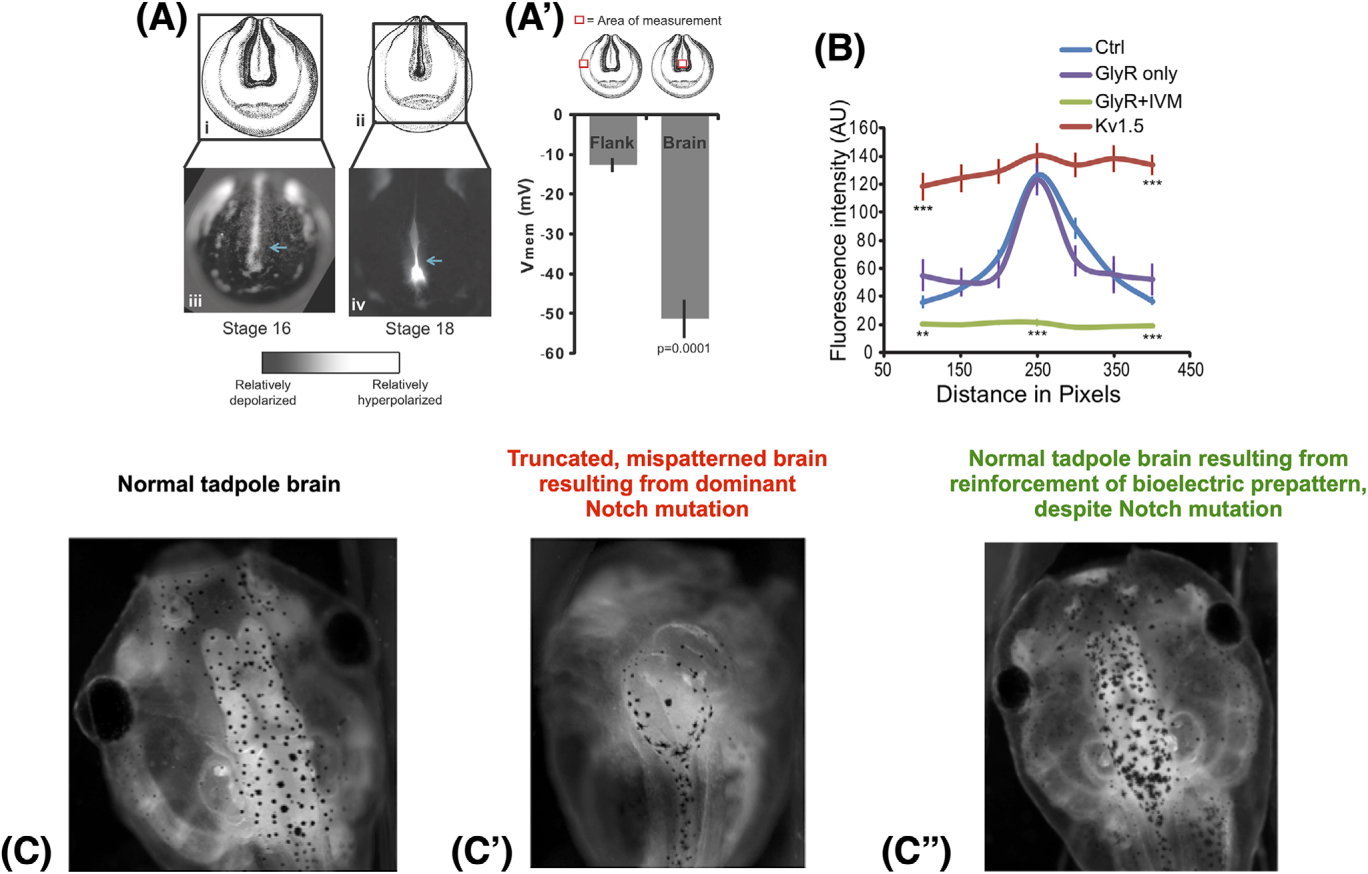
A context‐sensitive bioelectric intervention for birth defects. (A) The neurectoderm in early frog embryos bears a specific bioelectric pattern (quantified in A’) prior to forming the brain [124]. (B) That pattern is required for normal brain development, because equalizing the resting potential across the midline by bringing all the cells to the same high value (red) or the same low value (green), using chloride (GlyR) or potassium (Kv1.5) channel misexpression, results in severe brain defects: it is the *difference* between the cells at the left and right edge of the nascent brain field that is crucial—not a specific voltage number but a pattern. This pattern is disrupted by numerous teratogenic influences [125, 126, 127], including nicotine, alcohol, and mutation of the Notch gene. (C) The normal bilateral pattern of forebrain, midbrain, and hindbrain is severely disrupted after Notch mutation (C’): the forebrain is gone and the midbrain and hindbrain are reduced to fluid‐filled bubbles. These complex anatomical structures can be restored by reinforcing the bioelectric prepattern; this can be done, for example, by homogenously up‐regulating HCN2 channel activity, because this channel will sharpen the “bell curve” pattern by only hyperpolarizing the cells in the correct middle region, which are somewhat but not entirely depolarized as a result of the Notch mutation (C”). Images previously published in [124].

Cell collectives’ ability to read bioelectric state information can be used to repair birth defects in vertebrate embryos (Figure 5). Normal brain morphogenesis is determined by a specific bioelectric prepattern that is altered by chemical or genetic teratogens [124]. Forcing a return to the correct prepattern in the neural plate can correct brain morphology, gene expression, and learning capacity in animals exposed to alcohol, nicotine, or even mutations of the critical neurogenesis gene Notch [124, 125, 126]. Thus, at least some hardware defects (such as a dominant Notch mutation) can be fixed “in software” by a brief induced bioelectric pattern. The induction of this pattern does not require individual micromanagement of voltage state at every cell in the relevant region. Instead, a voltage‐sensitive ion channel—HCN2—can be activated, which causes different changes in depolarized and hyperpolarized cells. In effect, it is a “sharpen filter” for the bioelectric pattern that was blurred by the teratogens, establishing crisp lines between developmental compartments and leading to normal morphogenesis. This context‐sensitive property of HCN2 is the first step toward interventions that push complexity off the scientist and onto the system itself, prompting a primitive form of decision‐making within the system to communicate a complex goal.

The third mode of bioelectric network function encapsulates complex organ‐building cascades and binds them to specific voltage state triggers. One example is a very simple sodium flux to trigger the regeneration of a tail or limb in scenarios where they would not normally regenerate [128]. Another is the prepattern that determines the polarity of the planarian head‐tail axis [129]. Altering the bioelectric pattern for just a few hours can convert regenerating planaria into a 2‐headed form [130, 131]. Remarkably, the bioelectric circuit is a true memory because once flipped into a 2‐head state, it is permanent: 2‐headed worms will continue to regenerate with two heads in perpetuity without further manipulation [132]. This is not reflected by any genetic changes: it is an example of non‐genetic inheritance of morphology (previously shown only in single‐cell organisms [133, 134], though it also occurs in trophic memory of deer antler regeneration [135, 136]). Beyond reiterating the capacity for modular control, the two‐headed planarian is an example of rewriting the memory information that encodes the target morphology.

## 5 Cancer and the Scaling of the Self

Cancer is a highly complex, heterogeneous systemic disorder [137, 138, 139, 140]. Current approaches focus on genetic damage—a perspective in which irrevocably broken cells must be killed via application of toxic chemotherapies or immunotherapeutics [1]. This focus on cell cycle checkpoints and dysregulated molecular pathways predicts that animals with ready access to large numbers of plastic, undifferentiated, proliferative cells should be especially prone to cancer. In fact, it is often speculated that human bodies’ limited regenerative potential is an evolutionary tradeoff to limit the cancer burden in such a relatively long‐lived animal. But if so, what to make of the fact that animals such as planaria and salamanders are both highly regenerative and cancer resistant? An important clue to this apparent paradox is provided by the striking observation that regenerative [141, 142] and embryonic [68, 143] environments can reprogram cancer.

Perhaps a better question would be not why cancer occurs, but why is there ever anything but cancer—why do cells cooperate to build complex structures in the first place? Focusing on the initial state of all cells as highly proliferative unicellular organisms leads to a view of cancer as a breakdown of the mechanisms of multicellularity [144, 145, 146, 147]. But it is more than simply growth inhibition by neighbors. A recent theory of cancer [148, 149, 150] focuses on the concept of the cognitive light cone: the size of homeodynamic goal states that any active system can pursue. Consider an amoeba: all of its physiological, transcriptional, and metabolic goal states are limited to a very small spatial diameter, with very limited memory and predictive capacity (time horizons). Everything this cell does is in service to tiny goal states, and everything outside of this horizon is considered external environment, at the expense of which the cell may survive. What happens during evolution and developmental morphogenesis is an enormous scaling of this cognitive light cone. The cells belonging to a salamander limb are working on grandiose goals—achieving a complex structure of a highly specific size and form. It is a goal in the sense that when deviated from this attractor, the cells will rapidly work hard to get back to it (by rebuilding) and then stop when it is achieved. Compared to the amoeba, the salamander limb cellular collective's goal is much more complex, with a much larger spatiotemporal horizon.

Thus, we can view cancer from the perspective of basal cognition as a pathological rolling back to the smaller cognitive light cones of the deep past. This has already been confirmed for transcriptional profiles, as proposed in the atavistic theory of cancer [151, 152, 153], and has been extensively discussed in the literature addressing the inadequacies of the mutation theory of cancer [154, 155, 156, 157, 158]. But more specifically, the cognitive light cone model suggests that cancer cells are not more selfish; they just have smaller selves. They begin using action loops with much smaller local goals, in effect shrinking the boundary between self and world, reverting to an identity where the rest of the body, including neighboring cells, is treated as an external environment, outside the set of variables that must be maintained in desired ranges.

Many things, including but not limited to mutations, can kickstart the transformation process in which cells physiologically disconnect from the tissue‐level network. For example, a long period of stress can cause cells to close off gap junctions to prevent bystander toxicity effects. Each reduction in physiological connectivity makes it easier for cells to perform computations at the local level. This makes it easier for cells to close off gap junctions even more, driving a positive feedback loop—a cycle of dissociation and isolation that makes it ever harder to achieve large‐scale cell collective goals (maintaining complex anatomical structure). This picture is consistent with the known early steps of transformation involving gap junction closure [159, 160] and the seemingly paradoxical tumors induced by geometric barriers between tissues even when those barriers consist of materials that themselves are not carcinogenic [161, 162, 163].

This “changing boundary of the Self” model reinforces the theme of divergence between the DNA‐specified hardware and the physiological software that drives outcomes and makes strong predictions for a research agenda. If cancer is a failure of cognitive glue mechanisms that normally bind cells to common paths through morphospace, then targeting these mechanisms should enable: (a) detecting incipient cancer by monitoring cell physiological connectivity; (b) induction of cancer in genetically normal cells by physiological stimuli; and (c) normalization of cancer despite genetic defects. As described above, bioelectricity [21, 112] functions as the cognitive glue that scales up the cognitive light cone for navigation in anatomical space by cell collectives. Ion channel genes are increasingly recognized as oncogenes and ion channel drugs as potential electroceuticals [164, 165, 166, 167, 168, 169, 170, 171, 172], with increasing recognition that bioelectrical parameters are important in cancer initiation and metastasis [173, 174]. It has now been shown that (a) cancer induced by human oncogenes in vivo can be detected early by bioelectrical imaging [175], (b) transient perturbation of bioelectrical and serotonergic signaling among cells can induce a melanoma‐like phenotype in the absence of carcinogens, oncogenes, or DNA damage [176, 177, 178], and (c) tumors induced by powerful human oncogenes can be prevented and normalized by managing their bioelectric state [175, 179, 180, 181]. This ability to reinflate the cognitive light cone of cells shows that physiological information processing, not genetic hardware, dominates outcomes and suggests a clinical roadmap quite different from the chemotherapy that dominates the field today [165, 168, 182].

## 6 Applications and a Roadmap Toward a Radical Regenerative Medicine

**FIGURE 6:**
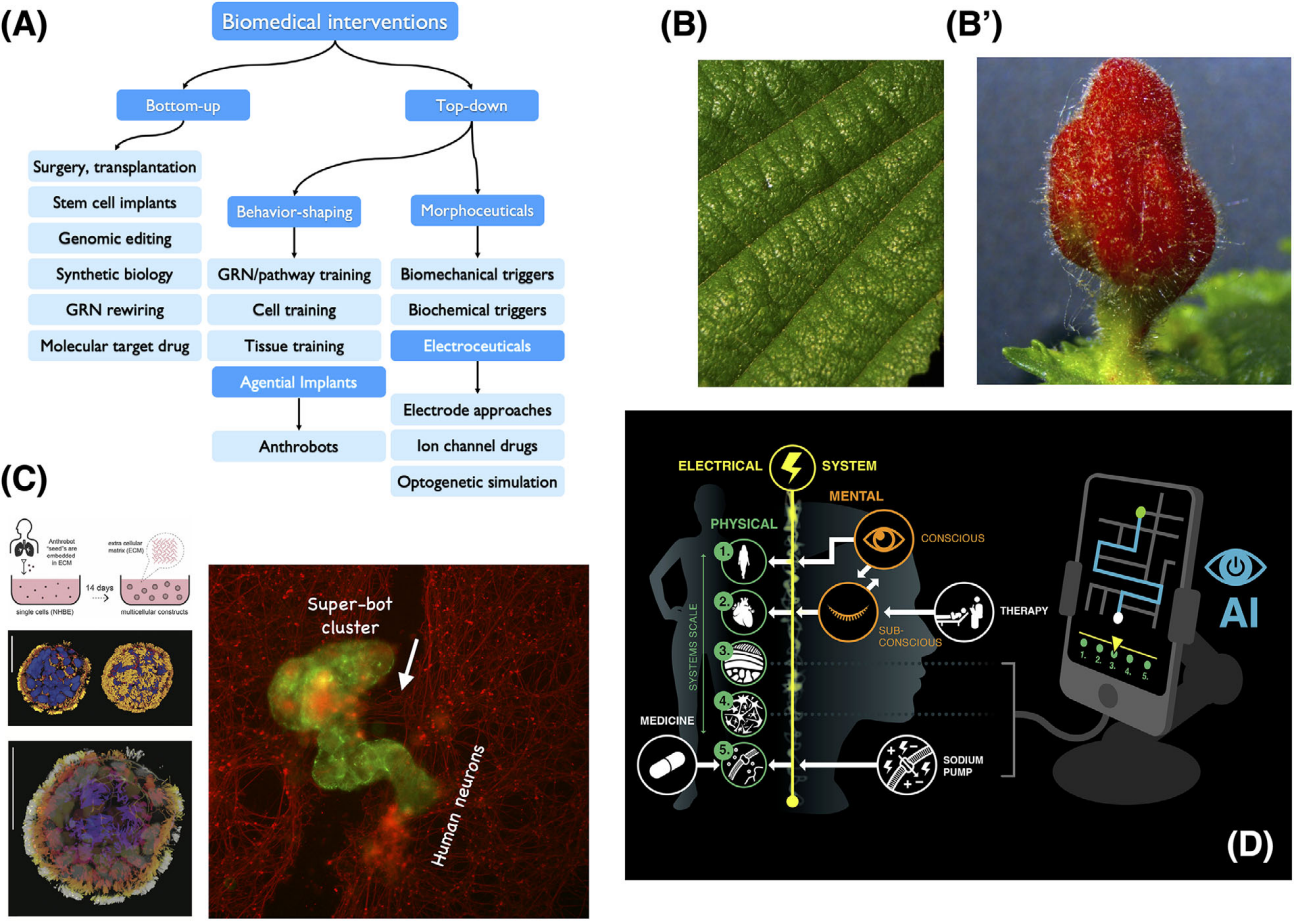
The future of biomedicine. (A) Biomedical interventions can be grouped into two major categories. The bottom‐up ones seek to directly manage hardware though surgery, transplantation, modification/creation of transcriptional circuits, and drugs that force specific pathway states. The top‐down approach, which is only now emerging in allopathic medicine, consists of attempts to signal to, communicate with, and change the internal memory content of the living material. Ways to control the agential material of the body include: shaping the behavior of pathways, cells, and tissues through training signals (rewards and punishments), deployment of interventions which are themselves not low‐agency drugs or materials but biobots or smart implants with goal‐directed loops and capacity to control cellular decision‐making, and modalities (drugs, light, etc.) which modify the course of complex morphogenesis by serving as triggers (for example for organ‐building cascades) or other ways to impact their navigation in morphospace. A subset of these morphoceuticals are electroceuticals, which use the bioelectric interface to communicate goal state information to tissues via ion channel and gap junctional modulation. Image previously published in [183]. (B) The capability of cellular collectives is not obvious from the default morphology, nor are “developmental constraints” (which may be more constraints in our thinking than in the traversal of the option space by a given system). The same cellular hardware can be pushed into novel configurations by specific prompts, which evolution exploits this routinely, using developmental modules, triggers, and setpoint‐signals [56, 184, 185, 186]. This is how biological systems control their own parts, but this strategy has not gone un‐noticed by parasites that exploit the competencies of their target material. The normal leaf architecture (B) becomes something completely different (B’)—a patterned gall—when prompted by a non‐human bioengineer: an insect parasite that hacks the competencies of the leaf cells. Interestingly, the sophistication of the galls produced matches the overall complexity level of the parasite—on a spectrum from virus to nematode to insect, the most advanced prompter is able to induce the most complex galls [187]; this bodes well for human bioengineers, if we can commit to the task of improving our bioprompting abilities. (C) Anthrobots are motile, self‐assembling constructs created from human tracheal epithelial cells [188]. Not only do the cells from adult patients form a totally new, functional shape but they also reveal interesting and unpredictable capabilities (E’), such as gathering into a super‐bot cluster (green) and healing injuries in cultured human iPSC‐derived neurons (red). White arrow points to the healed portion under the bots. Images previously published in [188]. (D) Future progress can be envisaged where AI‐augmented algorithms can intervene anywhere on the continuum of the body, from high‐level mental constructs in the brain to control structures within the collective intelligence of body cells. Such an AI can act as a translator of bioelectric and other information, mimicking the effects seen in “mind‐body medicine” where information crosses levels from the human personality's cognitive intent to the downstream changes of biochemical states. Image by Jeremy Guay of Peregrine Creative.

The competencies described above reveal new fundamental principles for biomedical discovery. Biomedicine becomes more about communication and collaboration with an unconventional agent and less about rewiring the molecular hardware to force specific phenotypic states. Crucially, categorical distinctions between molecular pathways (as “real” hardware targets) and cognitive content (memories, world models) are dissolved [54, 101]; ancient questions on the relationship of mind and matter are not merely philosophical but of immense practical urgency. While neuroscience has long grappled with the rich spectrum between organic versus psychic disease, somatic medicine has too long grappled under the assumption that all its challenges can be resolved at the organic end. Below are a few examples of specific research programs that could extend and distinguish future medicine from the status quo by better navigating the space of the possible in ways not accessible to purely bottom‐up design of interventions (Figure 6A,B).

### 6.1 Top‐Down Control Exploits Agential Materials by Pushing Complexity from the Engineer onto the System Itself

One feature of integrated large‐scale selves is that control signals are not local; the benefit of bioelectric information networks, from bacterial biofilms [105, 189] to brains [190, 191, 192, 193], is that they integrate across space and time. The causal structure of multi‐scale order in biology often means that diagnostics and interventions can be deployed far from the cells in question. Examples include planarian regeneration [194], the rapid change of voltage profile of a knee joint when the *contralateral* leg is amputated (Figure 4E, [122]), and the ability to control brain patterning [127, 195] and tumor incidence [180, 181] by modulating the bioelectric pattern of cells on the other side of the body.

Importantly, integrated control systems go beyond the typical examples of cells integrated into tissues. For example, the hypothesis of scale‐free biology [196] has led to the discovery that individual embryos in large groups form a kind of “hyperembryo” with its own unique transcriptome and an ability to solve teratogenic challenges far better than individual embryos (or small groups) exhibit [197]. Other recent work has identified social metabolic control of immune cells [198]. Short‐term opportunities targeting these kinds of phenomena, for example, by learning to fake the cross‐embryo morphogenetic assistance effect within one organism and deploying it in biomedical settings, are just a part of a much bigger effort of learning to communicate with, and thus control, larger levels of organization that are not apparent from molecular‐biology perspectives [14, 15].

Another aspect of biological agential material that simplifies therapeutic targeting is their context‐sensitivity with respect to interpreting stimuli. For example, tail regeneration is induced in tadpoles by bathing the entire animal in the bioelectric modifying drug, because only the wound cells are paying attention to the signal [128]. Similarly, only ectopic optic nerve (and not the normal nerves in the head) respond to drug‐based signals present systemically [199]. Even more remarkably, the exact same electroceutical—monensin—triggers tails in tail wounds and legs at leg wounds: the specificity in all of these cases is in the cell collective that responds to the stimulus [128, 200]. Thus, the intervention does not need to micromanage the spatial properties of the reagent, if we understand the circumstances under which the cells know what to do.

More broadly, the ability of cell groups to navigate anatomical and transcriptional space toward specific, encoded goals suggests that one powerful way to achieve complex goals is to rewrite the setpoints, as described above for planarian regeneration. Modifying setpoints as opposed to forcing specific states avoids the compensatory regulation described above for the multiple ectopic nascent eyes. This approach gets the system's “buy‐in” as to the correct state, avoiding triggering resistive responses because it makes the intervention appear as if it originated *within* the system itself.

While prepatterns of resting membrane potential are very convenient control knobs for complex downstream modules [90], bioelectricity is not the only modality. Proto‐cognitive competencies exist below the cell level, in the learning and optimization capabilities of molecular networks; thus, precisely timed stimuli (a.k.a., dynamiceuticals [201]) offer the possibility of benefitting from, or shutting down, cellular memories of past physiological events [23, 24]. Thus, the roadmap of the future includes not merely drug discovery, but *behavioral* discovery—we must learn to search the space of behavior‐shaping signals that motivate cells to adopt desired phenotypes. Many drugs eventually fail because the system finds a way around the intervention that seemed efficacious in 1st‐order modeling [202]. Second‐order interventions include: patterns of stimuli designed via behavioral control strategies (drug conditioning, training molecular and cellular components for desired outcomes using reward and punishment) [17], drugs that target homeostatic control loops, such as perhaps semaglutide [203, 204, 205], and anti‐cancer strategies that seek to reinforce cellular connections to the collective [149, 206]. Third‐order interventions could include psychedelics or plastogens to directly control the self‐models (and their plasticity) in tissue contexts [207, 208] and nootropics to help cells find better solutions to stressors.

Beyond new ways to use drugs to communicate with cells lie more complex agential interventions: reagents that are themselves context‐sensitive. This includes the HCN2 channel described above, which can be deployed systemically because the channel itself distinguishes cells in different physiological states and thus can sharpen voltage prepatterns (leading to a repair of birth defects) by selectively acting on depolarized cells [125, 126]. The next level of agential interventions are cellular constructs such as patient‐derived personalized biobots (Figure 6C), which can move autonomously and dynamically interact with their living or abiotic microenvironment, with context‐sensitive and tissue‐hacking competencies remaining to be discovered. These have been shown to induce repair of neural cells in vitro [188] and bile ducts [209]. The use of bespoke, personalized, patient‐derived biobots to exert repair within the body without need for genetic editing or immunosuppression is a major opportunity for biomedicine, as these share with the body a molecular understanding of inflammation, damage, cancer, microbiome, and health. Cell‐based biobots inherently contain a myriad of sensors and decision‐making machinery accumulated during a billion years of evolution and far beyond anything our nanotechnology can produce today.

### 6.2 A Better Understanding of Disease: From Molecular Markers to Physiological Patterns

One thing that needs to be expanded is the notion of “disease” beyond specific molecular markers/events to an understanding of undesired systemic states as learned attractors and dynamic patterns within physiological and other spaces [210, 211]. For example, the apparent inability of limbs to regenerate without neurons is *learned*—it is not an innate limitation but rather a kind of “nerve addiction” [212, 213, 214]. What other disease states and limitations of healthy functioning are the direct result of learned priors by cells and molecular networks?

The next stage of advances will go beyond conventional dynamical systems theory approaches to systems medicine by including not just a view of network states as passive features of a complex landscape but as *patterns within a proto‐cognitive system* that might themselves have minimal decision‐making and computational competency to facilitate their own persistence (in the same way that depressive and repetitive thoughts enact niche construction on the neural hardware of the brain to make it easier for such thoughts to exist and amplify [215]).

Given the many kinds of persistent, coherent, self‐reinforcing patterns of energy and information seen in dynamical systems theory and physics (solitons, autowaves, etc. [216, 217, 218, 219]), could some disease conditions be those kinds of persistent quasi‐objects—regions of transcriptional, physiological, or anatomical space that exist and exert causal power despite the fact that they are not classical *objects*? These have been found in physiological media, for example, as domain walls [220]—bioelectrical patterns propagating through homogenous tissue, and the bioelectric prepatterns discussed above are examples of these in vivo, as are “mirror foci” in the brain—epilepsy‐triggering physiological states that exist in a brain hemisphere opposite from the one that actually sustained damage [221, 222, 223, 224, 225, 226, 227]—a pernicious natural process that is the converse of bioengineers’ attempts to repair bioelectric state by modulating remote regions [127, 180, 181, 195]. Packets of stress, setpoints, self‐models, and estimates of safety may be agents in our body that live in physiological, metabolic, and transcriptional state space, impacting health and disease as their mental counterparts do for mental health.

Could some of these patterns within the cellular collective intelligence be addressed in the same way as harmful, persistent thoughts are treated within the neural cognitive system of patients [53, 228, 229, 230]? Some diseases may be due to perceptual illusions, sensory or attention deficits, mistaken beliefs, excessive insecurity, or harmful self‐models formed in tissue as a result of prior experiences and could be addressed by the powerful emerging tools of computational psychiatry and cognitive behavioral therapies, but aimed instead at the non‐neural intelligence in tissues and implemented in physiological and transcriptional spaces.

Conversely, not all disease may be caused by the addition of unwanted information patterns; we have previously proposed that aging is due to the degradation of important endogenous (bioelectric) pattern memories that are required throughout the lifespan to maintain tissue order [146, 147]. It is known that removing instructive signals, for example by denervation, can cause disorganization of mature tissues such as tongue papillae and, in general, render tissues much more susceptible to disorders of morphostasis such as cancer [231, 232, 233, 234]. Morphogenesis does not end when the body is complete—the Ship of Theseus is a good analogy for the body, consisting of the replacement policies in the somatic intelligence of the body that needs to make context‐sensitive repairs to a tight target specification. It is likely that solutions to the aging problem need to not only include rejuvenating signals at the cell level (e.g., Yamanaka factors) but also target the information scaffold needed to know what the new cells should do and where they should do it [235]. Some of that information could be imposed directly (by optogenetic or pharmacological stimuli), but some of it may be best implemented by facilitating the communication between the right kind of modules—letting cells talk to other cells, biobots, or next‐generation living “bandages.” In the case of cancer, 1st‐order treatments would be bioelectrical stimuli that force a hyperpolarized state [175, 181]; 2nd‐order treatments would facilitate gap junctional connections among cells or exposure to active morphogenetic cues (including non‐bioelectric ones) that are known to induce normalization [236, 237, 238]. Third‐order treatments may target the stress perception machinery in cells [239] to counteract the eventual shrinkage of the border between self and world that occurs in agents with continuous exposure to danger signals in their social milieu [150]. We can also go well beyond biomedicine in vivo, applying these same principles to bioengineering efforts to recreate organs and other needed living structures for transplantation or other purposes.

### 6.3 Eavesdropping on the Wisdom of the Body

Complementing the efforts to influence higher levels of biology (writing information into the system), the field must develop methods to query the insights that these higher levels have about their own function (reading information, at levels across the neuroscience spectrum ranging from physiological recording, behavioral analysis, and conversation).

Exploiting the problem‐solving capacities of cells and tissues is essential to unlock the promise of other, conventional technologies. For example, even when CRISPR becomes 100% reliable and specific for single‐gene diseases, genomic editing will still need to navigate a further barrier in order to develop applications for the control of growth and form: which genes must be edited to achieve a desired complex anatomical change? Planaria rapidly discern which of their tens of thousands of genes hold the answer to a novel stressor (barium) [66]. While it is not yet known how they do it, there exists a native mechanism for identifying the molecular affordances that can be activated to solve even unforeseen stressors [66]. Likewise, salamander cells call upon the right cell‐biological mechanism to make a kidney tubule out of many or just one cell [71, 100]. It should be possible to use imaging and computational interpretation filters to get cells (in situ or in bioengineered avatars) to *tell us* what steps they would take for a given situation and use it to guide gene therapy and pharmacological interventions. The technology that must be developed for this is simply the neural decoding [240, 241, 242, 243] system applied to non‐neural tissues.

More broadly, tools (such as AI applied to non‐invasive, multi‐modal physiological profiling across scales, Figure 6D) can provide a communications channel to biological sensors. In top‐down diagnostics, we might not try to read and interpret the status of specific markers but instead ask cells and tissues their perception of their neighbors and overall conditions. Not just surrogate site diagnostics at a distance [122], but using living components as the final layer of a classifier neural network to help interpret complex biological states as inputs. We could track stress levels and other aspects of behavior of cells, organoids, and biobots as they are exposed to patient tissues, to benefit from their built‐in ability to coarse‐grain and react to myriad physiological and biochemical parameters of their microenvironment. The combination of language models, living information filters, and physiomic profiling raises an intriguing possibility. If molecular states can be controlled through the linguistic interface in psychodermatology [244, 245, 246], perhaps similar techniques can be used to extract insight into health and disease processes. Spontaneous cases of novel, undiagnosed disease states being brought to clinician's attention via language [247], suggest that it may be possible to create tools that penetrate the normally tight virtualization (abstraction layers) used in biology and communicate bi‐directionally with organs, tissues, and cells.

## 7 Conclusion

Words and drugs have the same mechanism of action.

—Fabrizio Benedetti

A fundamental question about information in biology, especially setpoints, is “where is it encoded?” Physically, bodies consist of a functional hierarchy in which genes and the laws of chemistry determine the properties of molecular interaction, which in turn, with the laws of computation, determine the resulting behavior of cells, tissues, and organs as they self‐construct and repair toward complex anatomical outcomes. Philosophically, one can hold that the final result is encoded anywhere along that range of scales—from the basic laws of physics underlying everything that happens in the universe to the pattern memories that finally indicate a specific shape for a given organ. Practically, what matters is the *distance* between a given level of organization and the actionable information specifying outcomes one seeks to control. The distance between dynamic anatomy and the genetic sequence of proteins is enormous, due to the many active processes of morphogenesis that lie between them. The distance between the prepatterns (encoded for example in bioelectric states) and the anatomy is smaller. Crossing levels is very difficult, which is why we typically do not program our computers by tweaking the properties of silicon and copper. It is critical to identify the most proximal layer of description to the phenotypes in question and discover a set of tools to optimally manipulate the system at that level.

The major hypothesis discussed herein is a fundamental symmetry between mind and body, in terms of mechanisms and the functional causal architecture they implement. The implication of this perspective is that tools from behavioral neuroscience—ranging from electrophysiology to psychiatry—could be applicable outside the brain and its control of conventional behavior. Some of these tools have already been used to implement novel capabilities to control morphology in birth defects, regeneration, and cancer. Others remain speculative proposals whose value will be tested in forthcoming experimental approaches.

The key issue in any application in biomedicine, bioengineering, and physiological health/disease is: what is the optimal level of interaction (Figure 1A)? Training animals is more efficient than micromanaging nerves and muscles because it exploits the native top‐down control system that the body uses to manage low‐level molecular and cellular events toward whole‐body adaptive goals. Learning writes information into the medium much more efficiently than our clumsy interventions, and it behooves us to understand which layers offer what affordances to read and write information toward desired outcomes. In some cases, the most efficient targets will be molecular components. In others, it will be the higher‐order memories, goals, and self‐models of the cells and tissues. A crucial component of this strategy is an organicist perspective that treats the biology not as a simple machine and not just as a source of emergent complexity but as a multi‐scale society of agents with agendas. In psychiatry, the best predictor of success is the belief‐congruent approach in which the patient respects and feels aligned with the therapist. The somatic version of this is interventions that are perceived by cells and tissues as something they wanted to do in the first place—not micromanagement of symptoms but instead targeting deep control structures that represent the system's own goal states and memories, resulting in a “therapeutic alliance” [248]. The path to effective regenerative medicine is to take seriously the teleonomy that pervades living systems and harness it.

Albert Mason developed a remarkable practice of hypnodermatology [244, 249]—using commands filtered through the language interface to modify cellular behavior (now known as mind‐body medicine [250]). He eventually changed course and became a psychotherapist because he noted that his patients’ skin conditions would clear up, but they would develop problems elsewhere in their lives because, albeit at a higher level, he was still treating the manifestation of disease, not the underlying cause. Perhaps a similar path will play out in this field. Rewriting bioelectric patterns [116] and exploiting drug conditioning [54, 251, 252, 253] are higher‐level interventions than direct modulation of transcription factors and signaling proteins, but it may still be just a temporary crutch for an even higher‐level semantic interface. Top‐level executive mental constructs can cause ion flows in muscle (voluntary motion in the pursuit of career goals, for example). If bioelectricity transduces mental content into changes in biochemical events, and bioelectrical signals can induce regeneration [113, 254] and cancer normalization [148], is it possible that eventually, we will be able to go directly from mental states to anatomical outcomes, skipping the intermediate components described above? This, of course, has been suggested by alternative health practitioners for a very long time, in terms of their emphasis on the power of the mind for healing. But moving beyond theoretical claims toward reliable universal regenerative effects requires a lot of rigorous research to flesh out the connection across therapeutic levels via available technologies. Future medicine may look a lot more like psychiatry than it does like chemistry, because in the end, the real mind‐body medicine may have to target the many minds operating within the body, not just the mind of the “patient” that normally commandeers our attention. At stake are truly transformative applications ranging from repair of birth defects and injury to cancer reprogramming, bioengineered organs, and the freedom of embodiment offered by effective rational control over growth and form.

## Conflicts of Interest

My laboratory has sponsored research agreements with Morphoceuticals and Astonishing Labs, two companies that operate in a space relevant to this paper.

## Data Availability

No new data was generated in this Perspective article.

## References

[1] A. Raza , The First Cell: And the Human Costs of Pursuing Cancer to the Last (New York: Basic Books, 2019).

[2] A. Tschantz , L. Barca , D. Maisto , C. L. Buckley , A. K. Seth , and G. Pezzulo , “Simulating Homeostatic, Allostatic and Goal‐Directed Forms of Interoceptive Control Using Active Inference,” Biological Psychology 169 (2022): 108266. 35051559 10.1016/j.biopsycho.2022.108266

[3] B. S. McEwen , “Stress, Adaptation, and Disease: Allostasis and Allostatic Load,” Annals of the New York Academy of Sciences 840 (1998): 33–44. 9629234 10.1111/j.1749-6632.1998.tb09546.x

[4] W. B. Cannon , The Wisdom of the Body (New York: Norton, 1932).

[5] W. B. Cannon , “Organization for Physiological Homeostasis,” Physiological Reviews 9 (1929): 399–431.

[6] D. Noble , “Genes and Causation,” Philosophical Transactions of the Royal Society A 366 (2008): 3001–3015. 10.1098/rsta.2008.0086 18559318

[7] D. Noble , “A Theory of Biological Relativity: No Privileged Level of Causation,” Interface Focus 2 (2012): 55–64. 23386960 10.1098/rsfs.2011.0067 PMC3262309

[8] J. L. Steenwyk , D. A. Opulente , J. Kominek , et al., “Extensive Loss of Cell‐Cycle and DNA Repair Genes in an Ancient Lineage of Bipolar Budding Yeasts,” PLoS Biology 17 (2019): e3000255. 31112549 10.1371/journal.pbio.3000255 PMC6528967

[9] F. Cebrià , T. Adell , and E. Saló , “Rebuilding a Planarian: From Early Signaling to Final Shape,” International Journal of Developmental Biology 62 (2018): 537–550. 29938765 10.1387/ijdb.180042es

[10] O. Nishimura , K. Hosoda , E. Kawaguchi , et al., “Unusually Large Number of Mutations in Asexually Reproducing Clonal Planarian Dugesia japonica,” PLoS ONE 10 (2015): e0143525. 26588467 10.1371/journal.pone.0143525 PMC4654569

[11] F. Baluška and M. Levin , “On Having no Head: Cognition throughout Biological Systems,” Frontiers in Psychology 7 (2016): 902. 27445884 10.3389/fpsyg.2016.00902 PMC4914563

[12] P. McMillen and M. Levin , “Collective Intelligence: A Unifying Concept for Integrating Biology across Scales and Substrates,” Communications Biology 7 (2024): 378. 38548821 10.1038/s42003-024-06037-4 PMC10978875

[13] C. Fields and M. Levin , “Competency in Navigating Arbitrary Spaces as an Invariant for Analyzing Cognition in Diverse Embodiments,” Entropy 24, no. 6 (2022): 819. 35741540 10.3390/e24060819 PMC9222757

[14] G. Pezzulo and M. Levin , “Top‐Down Models in Biology: Explanation and Control of Complex Living Systems above the Molecular Level,” Journal of the Royal Society, Interface 13 (2016): 20160555. 27807271 10.1098/rsif.2016.0555 PMC5134011

[15] G. Pezzulo and M. Levin , “Re‐Membering the Body: Applications of Computational Neuroscience to the Top‐Down Control of Regeneration of Limbs and Other Complex Organs,” Integrative Biology 7 (2015): 1487–1517. 26571046 10.1039/c5ib00221d PMC4667987

[16] J. Davies and M. Levin , “Synthetic Morphology With Agential Materials,” Nature Reviews Bioengineering 1 (2023): 46–59.

[17] C. I. Abramson and M. Levin , “Behaviorist Approaches to Investigating Memory and Learning: A Primer for Synthetic Biology and Bioengineering,” Communicative & Integrative Biology 14 (2021): 230–247. 34925687 10.1080/19420889.2021.2005863 PMC8677006

[18] M. Levin , “Technological Approach to Mind Everywhere: An Experimentally‐Grounded Framework for Understanding Diverse Bodies and Minds,” Frontiers in Systems Neuroscience 16 (2022): 768201. 35401131 10.3389/fnsys.2022.768201 PMC8988303

[19] J. Bongard and M. Levin , “Living Things Are Not (20th Century) Machines: Updating Mechanism Metaphors in Light of the Modern Science of Machine Behavior,” Frontiers in Ecology and Evolution 9 (2021): 650726.

[20] E. Lagasse and M. Levin , “Future Medicine: From Molecular Pathways to the Collective Intelligence of the Body,” Trends in Molecular Medicine 29, no. 9 (2023): 687–710. 37481382 10.1016/j.molmed.2023.06.007 PMC10527237

[21] M. Levin , “Bioelectric Networks: The Cognitive Glue Enabling Evolutionary Scaling from Physiology to Mind,” Animal Cognition 26 (2023): 1865. 37204591 10.1007/s10071-023-01780-3 PMC10770221

[22] W. James , The Principles of Psychology (New York: H. Holt and Company, 1890).

[23] S. Biswas , W. Clawson , and M. Levin , “Learning in Transcriptional Network Models: Computational Discovery of Pathway‐Level Memory and Effective Interventions,” International Journal of Molecular Sciences 24 (2022): 285. 36613729 10.3390/ijms24010285 PMC9820177

[24] S. Biswas , S. Manicka , and E. Hoel , and M. Levin , “Gene Regulatory Networks Exhibit Several Kinds of Memory: Quantification of Memory in Biological and Random Transcriptional Networks,” Iscience 24 (2021): 102131. 33748699 10.1016/j.isci.2021.102131 PMC7970124

[25] K. J. Friston , J. Daunizeau , J. Kilner , and S. J. Kiebel , “Action and Behavior: A Free‐Energy Formulation,” Biological Cybernetics 102 (2010): 227–260. 20148260 10.1007/s00422-010-0364-z

[26] K. Friston , “Life as We Know It,” Journal of the Royal Society, Interface /the Royal Society 10 (2013): 20130475. 10.1098/rsif.2013.0475 PMC3730701 23825119

[27] F. Baluska and A. S. Reber , “Cellular Sentience as the Primary Source of Biological Order and Evolution,” Bio Systems 218 (2022): 104694. 10.1016/j.biosystems.2022.104694 35595194

[28] P. Lyon , “The Biogenic Approach to Cognition,” Cognitive Process 7 (2006): 11–29. 10.1007/s10339-005-0016-8 16628463

[29] P. Lyon , “The Cognitive Cell: Bacterial Behavior Reconsidered,” Frontiers in Microbiology 6 (2015): 264. 25926819 10.3389/fmicb.2015.00264 PMC4396460

[30] J. P. Headrick , L. E. See Hoe , E. F. Du Toit , and J. N. Peart , “Opioid Receptors and Cardioprotection—‘Opioidergic Conditioning’ of the Heart,” British Journal of Pharmacology 172 (2015): 2026–2050. 25521834 10.1111/bph.13042 PMC4386979

[31] M. Kunecki , T. Roleder , J. Biernat , et al., “Opioidergic Conditioning of the Human Heart Muscle in Nitric Oxide‐Dependent Mechanism,” Advances in Clinical and Experimental Medicine: Official Organ Wroclaw Medical University 27 (2018): 1069–1073. 29912480 10.17219/acem/70192

[32] Y. E. Antebi , J. M. Linton , H. Klumpe , et al., “Combinatorial Signal Perception in the BMP Pathway,” Cell 170 (2017): 1184–1196 e24. 10.1016/j.cell.2017.08.015 PMC5612783 28886385

[33] L. J. Bugaj , G. P. O'Donoghue , and W. A. Lim , “Interrogating Cellular Perception and Decision Making With Optogenetic Tools,” Journal of Cell Biology 216 (2017): 25–28. 28003330 10.1083/jcb.201612094 PMC5223619

[34] A. Mitchell and W. Lim , “Cellular Perception and Misperception: Internal Models for Decision‐Making Shaped by Evolutionary Experience,” BioEssays 38 (2016): 845–849. 27461864 10.1002/bies.201600090 PMC4996742

[35] E. Dine , A. A. Gil , G. Uribe , C. P. Brangwynne , and J. E. Toettcher , “Protein Phase Separation Provides Long‐Term Memory of Transient Spatial Stimuli,” Cell Systems 6 (2018): 655–663 e5. 10.1016/j.cels.2018.05.002 PMC6023754 29859829

[36] M. Z. Wilson , P. T. Ravindran , W. A. Lim , and J. E. Toettcher , “Tracing Information Flow from Erk to Target Gene Induction Reveals Mechanisms of Dynamic and Combinatorial Control,” Molecular Cell 67 (2017): 757–769 e5. 10.1016/j.molcel.2017.07.016 PMC5591080 28826673

[37] B. Zakirov , G. Charalambous , R. Thuret , et al., “Active Perception During Angiogenesis: Filopodia Speed up Notch Selection of Tip Cells in Silico and in Vivo,” Philosophical Transactions of the Royal Society of London Series B: Biological Sciences 376 (2021): 20190753. 33550953 10.1098/rstb.2019.0753 PMC7934951

[38] K. Bentley and S. Chakravartula , “The Temporal Basis of Angiogenesis,” Philosophical Transactions of the Royal Society of London Series B: Biological Sciences 372 (2017): 20150522. 28348255 10.1098/rstb.2015.0522 PMC5379027

[39] K. Bentley , A. Philippides , and E. Ravasz Regan , “Do Endothelial Cells Dream of Eclectic Shape?” Developmental Cell 29 (2014): 146–158. 24780735 10.1016/j.devcel.2014.03.019

[40] P. Csermely , N. Kunsic , P. Mendik , et al., “Learning of Signaling Networks: Molecular Mechanisms,” Trends in Biochemical Sciences 45 (2020): 284–294. 32008897 10.1016/j.tibs.2019.12.005

[41] Y. Katz and W. Fontana , “Probabilistic Inference With Polymerizing Biochemical Circuits,” Entropy 24 (2022): 629. 35626513 10.3390/e24050629 PMC9140500

[42] Y. Katz , M. Springer , and W. Fontana . 2018. “Embodying Probabilistic Inference in Biochemical Circuits.” arXiv:1806.10161.

[43] A. Koseska and P. I. Bastiaens , “Cell Signaling as a Cognitive Process,” Embo Journal 36 (2017): 568–582. 28137748 10.15252/embj.201695383 PMC5331751

[44] G. Albrecht‐Buehler , “Is Cytoplasm Intelligent Too?” Cell & Muscle Motility 6 (1985): 1–21. 4039627 10.1007/978-1-4757-4723-2_1

[45] W. B. Miller Jr , F. Baluska , and A. S. Reber , “A Revised central Dogma for the 21st Century: All Biology Is Cognitive Information Processing,” Progress in Biophysics and Molecular Biology 182 (2023): 34–48. 37268025 10.1016/j.pbiomolbio.2023.05.005

[46] F. Baluska , W. B. Miller , and A. S. Reber , “Cellular and Evolutionary Perspectives on Organismal Cognition: From Unicellular to Multicellular Organisms,” Biological Journal of the Linnean Society 139, no. 4 (2022): 503–513.

[47] F. Baluska and W. B. Miller Jr , and A. S. Reber , “2021. Biomolecular Basis of Cellular Consciousness via Subcellular Nanobrains,” International Journal of Molecular Sciences 22, no. 5: 2545. 33802617 10.3390/ijms22052545 PMC7961929

[48] W. B. Miller Jr. , J. S. Torday , and F. Baluska , “The N‐Space Episenome Unifies Cellular Information Space‐Time Within Cognition‐Based Evolution,” Progress in Biophysics and Molecular Biology 150 (2020): 112–139. 31415772 10.1016/j.pbiomolbio.2019.08.006

[49] Y. Timsit and S. P. Gregoire , “Towards the Idea of Molecular Brains,” International Journal of Molecular Sciences 22 (2021): 11868. 34769300 10.3390/ijms222111868 PMC8584932

[50] F. Baluska , “Senomic View of the Cell: Senome versus Genome,” Communicative & Integrative Biology 11 (2018): 1–9. 10.1080/19420889.2018.1489184 PMC6132427 30214674

[51] S. A. Kauffman , “Control Circuits for Determination and Transdetermination,” Science 181 (1973): 310–318. 4198229 10.1126/science.181.4097.310

[52] S. A. Kauffman , The Origins of Order: Self Organization and Selection in Evolution (New York: Oxford University Press, 1993).

[53] K. J. Friston , K. E. Stephan , R. Montague , and R. J. Dolan , “Computational Psychiatry: The Brain as a Phantastic Organ,” Lancet Psychiatry 1 (2014): 148–158. 26360579 10.1016/S2215-0366(14)70275-5

[54] J. Mathews , A. J. Chang , L. Devlin , and M. Levin , “Cellular Signaling Pathways as Plastic, Proto‐Cognitive Systems: Implications for Biomedicine,” Patterns 4 (2023): 100737. 37223267 10.1016/j.patter.2023.100737 PMC10201306

[55] L. Shreesha and M. Levin , “Cellular Competency During Development Alters Evolutionary Dynamics in an Artificial Embryogeny Model,” Entropy 25 (2023): 131. 36673272 10.3390/e25010131 PMC9858125

[56] M. Levin , “Darwin's Agential Materials: Evolutionary Implications of Multiscale Competency in Developmental Biology,” Cellular and Molecular Life Sciences 80 (2023): 142. 37156924 10.1007/s00018-023-04790-z PMC10167196

[57] R. A. Watson , C. L. Buckley , R. Mills , and A. Davies . “Associative Memory in Gene Regulation Networks,” in Proceedings of the Artificial Life Conference XII , eds. H. Fellerman , M. Dörr , M. M. Hanczyc , L. L. Laursen , S. Maurer , D. Merkle , P.‐A. Monnard , K. Stoy , and S. Rasmussen , (Boston, MA: MIT Press, 2010), 194–201.

[58] R. A. Watson and E. Szathmary , “How Can Evolution Learn?” Trends in Ecology & Evolution 31 (2016): 147–157. 26705684 10.1016/j.tree.2015.11.009

[59] B. Hartl , S. Risi , and M. Levin , “Evolutionary Implications of Self‐Assembling Cybernetic Materials With Collective Problem‐Solving Intelligence at Multiple Scales,” Entropy 26 (2024): 532. 39056895 10.3390/e26070532 PMC11275831

[60] G. E. Hinton and J. Nowlan , “How Learning Can Guide Evolution,” Complex System 1 (1987): 495–502.

[61] P. L. Freddolino , J. Yang , A. Momen‐Roknabadi , and S. Tavazoie , “Stochastic Tuning of Gene Expression Enables Cellular Adaptation in the Absence of Pre‐Existing Regulatory Circuitry,” Elife 7 (2018): e31867. 29620524 10.7554/eLife.31867 PMC5919758

[62] P. L. Freddolino and S. Tavazoie , “Beyond Homeostasis: A Predictive‐Dynamic Framework for Understanding Cellular Behavior,” Annual Review of Cell and Developmental Biology 28 (2012): 363–384. 10.1146/annurev-cellbio-092910-154129 22559263

[63] H. I. Schreier , Y. Soen , and N. Brenner , “Exploratory Adaptation in Large Random Networks,” Nature Communications 8 (2017): 14826. 10.1038/ncomms14826 PMC5413947 28429717

[64] Y. Soen , M. Knafo , and M. Elgart , “A Principle of Organization Which Facilitates Broad Lamarckian‐Like Adaptations by Improvisation,” Biology Direct 10 (2015): 68. 26631109 10.1186/s13062-015-0097-y PMC4668624

[65] M. Elgart , O. Snir , and Y. Soen , “Stress‐Mediated Tuning of Developmental Robustness and Plasticity in Flies,” Biochimica Et Biophysica Acta 1849 (2015): 462–466. 25134463 10.1016/j.bbagrm.2014.08.004

[66] M. Emmons‐Bell , F. Durant , A. Tung , et al., “Regenerative Adaptation to Electrochemical Perturbation in Planaria: A Molecular Analysis of Physiological Plasticity,” Iscience 22 (2019): 147–165. 31765995 10.1016/j.isci.2019.11.014 PMC6881696

[67] L. N. Vandenberg , D. S. Adams , and M. Levin , “Normalized Shape and Location of Perturbed Craniofacial Structures in the Xenopus Tadpole Reveal an Innate Ability to Achieve Correct Morphology,” Developmental Dynamics 241 (2012): 863–878. 22411736 10.1002/dvdy.23770 PMC3428595

[68] B. Mintz and K. Illmensee , “Normal Genetically Mosaic Mice Produced from Malignant Teratocarcinoma Cells,” Proceedings of the National Academy of Sciences 72 (1975): 3585–3589. 10.1073/pnas.72.9.3585 PMC433040 1059147

[69] M. Rahbaran , E. Razeghian , M. S. Maashi , et al., “Cloning and Embryo Splitting in Mammalians: Brief History, Methods, and Achievements,” Stem Cells International 2021 (2021): 2347506. 34887927 10.1155/2021/2347506 PMC8651392

[70] A. K. Tarkowski , “Mouse Chimæras Developed from Fused Eggs,” Nature 190 (1961): 857–860. 13775333 10.1038/190857a0

[71] G. Fankhauser , “Maintenance of Normal Structure in Heteroploid Salamander Larvae, through Compensation of Changes in Cell Size by Adjustment of Cell Number and Cell Shape,” Journal of Experimental Zoology 100 (1945): 445–455. 21010861 10.1002/jez.1401000310

[72] W. A. Harris and V. Hartenstein , “Neuronal Determination Without Cell Division in Xenopus Embryos,” Neuron 6 (1991): 499–515. 1901716 10.1016/0896-6273(91)90053-3

[73] L. Zhang , C. Kendrick , D. Julich , and S. A. Holley , “Cell Cycle Progression Is Required for Zebrafish Somite Morphogenesis but Not Segmentation Clock Function,” Development 135 (2008): 2065–2070. 18480162 10.1242/dev.022673 PMC2923836

[74] J. Cooke , “Scale of Body Pattern Adjusts to Available Cell Number in Amphibian Embryos,” Nature 290 (1981): 775–778. 7219562 10.1038/290775a0

[75] A. Zarzosa , K. Grassme , E. Tanaka , et al., “Axolotls With an Under‐ or Oversupply of Neural Crest Can Regulate the Sizes of Their Dorsal Root Ganglia to Normal Levels,” Developmental Biology 394 (2014): 65–82. 25111151 10.1016/j.ydbio.2014.08.001

[76] J. Holtfreter , “Transformation of a Tail into a Limb or Gill‐like Structures,” Journal of Experimental Zoology 129 (1955): 623–648.

[77] N. Farinella‐Ferruzza , “The Transformation of a Tail into Limb after Xenoplastic Transplantation,” Experientia 15 (1956): 304–305.

[78] G. Serrano Najera and C. J. Weijer , “The Evolution of Gastrulation Morphologies,” Development 150 (2023): 200885. 10.1242/dev.200885 PMC10216749 37067451

[79] A. Voskoboynik , N. Simon‐Blecher , Y. Soen , et al., “Striving for Normality: Whole Body Regeneration through a Series of Abnormal Generations,” FASEB Journal 21 (2007): 1335–1344. 17289924 10.1096/fj.06-7337com

[80] A. Kirillova , G. Genikhovich , E. Pukhlyakova , A. Demilly , Y. Kraus , and U. Technau , “Germ‐Layer Commitment and Axis Formation in Sea Anemone Embryonic Cell Aggregates,” Proceedings of National Academy of Sciences 115 (2018): 1813–1818. 10.1073/pnas.1711516115 PMC5828576 29440382

[81] A. Rosello‐Diez , L. Madisen , S. Bastide , H. Zeng , and A. L. Joyner , “Cell‐Nonautonomous Local and Systemic Responses to Cell Arrest Enable Long‐Bone Catch‐up Growth in Developing Mice,” PLoS Biology 16 (2018): e2005086. 29944650 10.1371/journal.pbio.2005086 PMC6019387

[82] T. W. Hiscock , P. Tschopp , and C. J. Tabin , “On the Formation of Digits and Joints During Limb Development,” Developmental Cell 41 (2017): 459–465. 28586643 10.1016/j.devcel.2017.04.021 PMC5546220

[83] A. H. Huang , T. J. Riordan , B. Pryce , et al., “Musculoskeletal Integration at the Wrist Underlies the Modular Development of Limb Tendons,” Development 142 (2015): 2431–2441. 26062940 10.1242/dev.122374 PMC4510863

[84] C. Mehring , M. Akselrod , L. Bashford , et al., “Augmented Manipulation Ability in Humans With Six‐Fingered Hands,” Nature Communications 10 (2019): 2401. 10.1038/s41467-019-10306-w PMC6547737 31160580

[85] J. M. Hurle , M. A. Ros , Y. Ganan , D. Macias , M. Critchlow , and J. R. Hinchliffe , “Experimental Analysis of the Role of ECM in the Patterning of the Distal Tendons of the Developing Limb Bud,” Cell Differentiation and Development 30 (1990): 97–108. 2386888 10.1016/0922-3371(90)90078-b

[86] G. E. Little , G. Lopez‐Bendito , A. E. Runker , et al., “Specificity and Plasticity of Thalamocortical Connections in Sema6A Mutant Mice,” PLoS Biology 7 (2009): e98. 19402755 10.1371/journal.pbio.1000098 PMC2672616

[87] E. J. Slijper , “Biologic Anatomical Investigations on the Bipedal Gait and Upright Posture in Mammals—With Special Reference to a Little Goat Born Without Forelegs II,” Proceedings of the Koninklijke Nederlandse Akademie Van Wetenschappen 45 (1942): 407–415.

[88] E. M. P. Almazan , J. F. Ryan , and L. Rouhana , “Regeneration of Planarian Auricles and Reestablishment of Chemotactic Ability,” Frontiers in Cell and Developmental Biology 9 (2021): 777951. 34901022 10.3389/fcell.2021.777951 PMC8662385

[89] A. Rosenblueth and N. Wiener , and J. Bigelow , “Behavior, Purpose, and Teleology,” Philosophy of Science 10 (1943): 18–24.

[90] M. Levin and C. J. Martyniuk , “The Bioelectric Code: An Ancient Computational Medium for Dynamic Control of Growth and Form,” Bio Systems 164 (2018): 76–93. 10.1016/j.biosystems.2017.08.009 PMC10464596 28855098

[91] J. R. Stone , “The Spirit of D'arcy Thompson Dwells in Empirical Morphospace,” Mathematical Biosciences 142 (1997): 13–30. 9125858 10.1016/s0025-5564(96)00186-1

[92] D. M. Raup and A. Michelson , “Theoretical Morphology of the Coiled Shell,” Science 147 (1965): 1294–1295. 17790826 10.1126/science.147.3663.1294

[93] K. G. Sullivan , M. Emmons‐Bell , and M. Levin , “Physiological Inputs Regulate Species‐Specific Anatomy During Embryogenesis and Regeneration,” Communicative & Integrative Biology 9 (2016): e1192733. 27574538 10.1080/19420889.2016.1192733 PMC4988443

[94] M. Emmons‐Bell , F. Durant , J. Hammelman , et al., “Gap Junctional Blockade Stochastically Induces Different Species‐Specific Head Anatomies in Genetically Wild‐Type Girardia Dorotocephala Flatworms,” International Journal of Molecular Sciences 16 (2015): 27865–27896. 26610482 10.3390/ijms161126065 PMC4661923

[95] C. Fields , J. Bischof , and M. Levin , “Morphological Coordination: A Common Ancestral Function Unifying Neural and Non‐Neural Signaling,” Physiology 35 (2020): 16–30. 31799909 10.1152/physiol.00027.2019

[96] T. O'Brien , J. Stremmel , L. Pio‐Lopez , P. McMillen , C. Rasmussen‐Ivey , and M. Levin , “Machine Learning for Hypothesis Generation in Biology and Medicine: Exploring the Latent Space of Neuroscience and Developmental Bioelectricity,” Digital Discovery 3 (2024): 249–263.

[97] K. Friston , M. Levin , B. Sengupta , and G. Pezzulo , “Knowing One's Place: A Free‐Energy Approach to Pattern Regulation,” Journal of the Royal Society, Interface 12 (2015): 20141383. 25788538 10.1098/rsif.2014.1383 PMC4387527

[98] P. Lyon and F. Kuchling , “Valuing What Happens: A Biogenic Approach to Valence and (Potentially) Affect,” Philosophical Transactions of the Royal Society of London Series B: Biological Sciences 376 (2021): 20190752. 33487109 10.1098/rstb.2019.0752 PMC7935054

[99] F. Heylighen . 2023. “Relational Agency: A New Ontology for co‐Evolving systems,” in Evolution ‘on Purpose’: Teleonomy in Living Systems, eds. P. Corning , S. A. Kauffman , D. Noble , and J. A. Shapi , et al. (Cambridge, MA: MIT Press, 2023), 79–104.

[100] G. Fankhauser , “The Effects of Changes in Chromosome Number on Amphibian Development,” Quarterly Review of Biology 20 (1945): 20–78.

[101] M. Levin , “Self‐Improvising Memories: A Perspective on Memories as Agential, Dynamically‐Reinterpreting Cognitive Glue,” Entropy 26 (2024): 481. 38920491 10.3390/e26060481 PMC11203334

[102] R. Law and M. Levin , “Bioelectric Memory: Modeling Resting Potential Bistability in Amphibian Embryos and Mammalian Cells,” Theoretical Biology and Medical Modelling 12 (2015): 22. 26472354 10.1186/s12976-015-0019-9 PMC4608135

[103] D. E. Koshland , “The Bacterium as a Model Neuron,” Trends in Neurosciences 6 (1983): 133–137.

[104] A. Prindle , J. Liu , M. Asally , S. Ly , J. Garcia‐Ojalvo , and G. M. Süel , “Ion Channels Enable Electrical Communication in Bacterial Communities,” Nature 527 (2015): 59–63. 26503040 10.1038/nature15709 PMC4890463

[105] R. Martinez‐Corral , J. Liu , A. Prindle , G. M. Suel , and J. García‐Ojalvo , “Metabolic Basis of Brain‐Like Electrical Signalling in Bacterial Communities,” Philosophical Transactions of the Royal Society of London Series B: Biological Sciences 374 (2019): 20180382. 31006362 10.1098/rstb.2018.0382 PMC6553584

[106] F. Kuchling , K. Friston , G. Georgiev , and M. Levin , “Morphogenesis as Bayesian Inference: A Variational Approach to Pattern Formation and Control in Complex Biological Systems,” Physics of Life Reviews 33 (2020): 88–108. 31320316 10.1016/j.plrev.2019.06.001

[107] G. Pezzulo , J. LaPalme , F. Durant , and M. Levin , “Bistability of Somatic Pattern Memories: Stochastic Outcomes in Bioelectric Circuits Underlying Regeneration,” Philosophical Transactions of the Royal Society of London Series B: Biological Sciences 376 (2021): 20190765. 33550952 10.1098/rstb.2019.0765 PMC7935058

[108] M. Neuhof , M. Levin , and O. Rechavi , “Vertically‐ and Horizontally‐Transmitted Memories—The Fading Boundaries between Regeneration and Inheritance in planaria,” Biology Open 5 (2016): 1177–1188. 27565761 10.1242/bio.020149 PMC5051648

[109] S. Grossberg , “Communication, Memory, and Development,” in Progress in Theoretical Biology, eds. R. Rosen and F. Snell (Boston, MA: Boston University, 1978), 183–232.

[110] P. Srivastava , A. Kane , C. Harrison , and M. Levin , “A Meta‐Analysis of Bioelectric Data in Cancer, Embryogenesis, and Regeneration,” Bioelectricity 3 (2020): 42–67. 10.1089/bioe.2019.0034 PMC8370481 34476377

[111] L. F. George , S. J. Pradhan , D. Mitchell , et al., “Ion Channel Contributions to Wing Development in Drosophila Melanogaster,” G3 Genes|Genomes|Genetics 9 (2019): 999–1008. 30733380 10.1534/g3.119.400028 PMC6469425

[112] E. Bates , “Ion Channels in Development and Cancer,” Annual Review of Cell and Developmental Biology 31 (2015): 231–247. 10.1146/annurev-cellbio-100814-125338 26566112

[113] M. P. Harris , “Bioelectric Signaling as a Unique Regulator of Development and Regeneration,” Development 148 (2021). 10.1242/dev.180794 PMC8180260 33999994

[114] R. Nuccitelli , “Endogenous Electric Fields in Embryos During Development, Regeneration and Wound Healing,” Radiation Protection Dosimetry 106 (2003): 375–383. 14690282 10.1093/oxfordjournals.rpd.a006375

[115] K. R. Robinson and M. A Messerli . 1996. “Electric Embryos: The Embryonic Epithelium as a Generator of Developmental information.” in Nerve Growth and Guidance, ed. C. D. McCaig (London: Portland Press, 1996), 131–150.

[116] M. Levin , “Bioelectric Signaling: Reprogrammable Circuits Underlying Embryogenesis, Regeneration, and Cancer,” Cell 184 (2021): 1971–1989. 33826908 10.1016/j.cell.2021.02.034

[117] S. Sundelacruz , M. Levin , and D. L. Kaplan , “Role of Membrane Potential in the Regulation of Cell Proliferation and Differentiation,” Stem Cell Reviews and Reports 5 (2009): 231–246. 19562527 10.1007/s12015-009-9080-2 PMC10467564

[118] L. N. Vandenberg , R. D. Morrie , and D. S. Adams , “V‐ATPase‐Dependent Ectodermal Voltage and Ph Regionalization Are Required for Craniofacial Morphogenesis,” Developmental Dynamics 240 (2011): 1889–1904. 21761475 10.1002/dvdy.22685 PMC10277013

[119] J. M. Statland , R. Tawil , and S. L Venance . 1993. “Andersen‐Tawil Syndrome.” in GeneReviews(R), R. A. Pagon , M. P. Adam , H. H. Ardinger , S. E. Wallace , et al., eds. (Seattle, WA: University of Washington, 1993). 20301441

[120] V. P. Pai , S. Aw , T. Shomrat , J. M. Lemire , and M. Levin , “Transmembrane Voltage Potential Controls Embryonic Eye Patterning in Xenopus laevis,” Development 139 (2012): 313–323. 22159581 10.1242/dev.073759 PMC3243095

[121] M. Fisher , C. James‐Zorn , V. Ponferrada , et al., “Xenbase: key features and resources of the Xenopus model organism knowledgebase,” Genetics 224 (2023): iyad018. 36755307 10.1093/genetics/iyad018 PMC10158840

[122] S. M. Busse , P. T. McMillen , and M. Levin , “Cross‐Limb Communication During Xenopus Hindlimb Regenerative Response: Non‐Local Bioelectric Injury Signals,” Development 145, no. 19 (2018): 16420. 10.1242/dev.164210 30126906

[123] R. L. Chow , C. R. Altmann , R. A. Lang , and A. Hemmati‐Brivanlou , “Pax6 induces Ectopic Eyes in a Vertebrate,” Development S126 (1999): S4213–S4222. 10.1242/dev.126.19.4213 10477290

[124] V. P. Pai , J. M. Lemire , J. F. Pare , G. Lin , Y. Chen , and M. Levin , “Endogenous Gradients of Resting Potential Instructively Pattern Embryonic Neural Tissue via Notch Signaling and Regulation of Proliferation,” Journal of Neuroscience 35 (2015): 4366–4385. 25762681 10.1523/JNEUROSCI.1877-14.2015 PMC4355204

[125] V. P. Pai , A. Pietak , V. Willocq , B. Ye , N.‐Q. Shi , and M. Levin , “HCN2 Rescues Brain Defects by Enforcing Endogenous Voltage Pre‐Patterns,” Nature Communications 9 (2018): 998. 10.1038/s41467-018-03334-5 PMC5843655 29519998

[126] V. P. Pai and M. Levin , “HCN2 Channel‐Induced Rescue of Brain, Eye, Heart and Gut Teratogenesis Caused by Nicotine, Ethanol and Aberrant Notch Signalling,” Wound Repair and Regeneration 30, no. 6 (2022): 681–706. 35662339 10.1111/wrr.13032

[127] V. P. Pai , J. Cervera , S. Mafe , V. Willocq , E. K. Lederer , and M. Levin , “HCN2 Channel‐INDUCED Rescue of Brain Teratogenesis via Local and Long‐Range Bioelectric Repair,” Frontiers in Cellular Neuroscience 14 (2020): 136. 32528251 10.3389/fncel.2020.00136 PMC7264377

[128] A. S. Tseng , W. S. Beane , J. M. Lemire , A. Masi , and M. Levin , “Induction of Vertebrate Regeneration by a Transient Sodium Current,” Journal of Neuroscience 30 (2010): 13192–13200. 20881138 10.1523/JNEUROSCI.3315-10.2010 PMC2965411

[129] W. S. Beane , J. Morokuma , D. S. Adams , and M. Levin , “A Chemical Genetics Approach Reveals H,K‐ATPase‐Mediated Membrane Voltage Is Required for Planarian Head Regeneration,” Chemistry & Biology 18 (2011): 77–89. 21276941 10.1016/j.chembiol.2010.11.012 PMC3278711

[130] F. Durant , J. Morokuma , C. Fields , K. Williams , D. S. Adams , and M. Levin , “Long‐Term, Stochastic Editing of Regenerative Anatomy via Targeting Endogenous Bioelectric Gradients,” Biophysical Journal 112 (2017): 2231–2243. 28538159 10.1016/j.bpj.2017.04.011 PMC5443973

[131] F. Durant , J. Bischof , C. Fields , et al., “The Role of Early Bioelectric Signals in the Regeneration of Planarian Anterior/Posterior Polarity,” Biophysical Journal 116 (2019): 948–961. 30799071 10.1016/j.bpj.2019.01.029 PMC6401388

[132] N. J. Oviedo , J. Morokuma , P. Walentek , et al., “Long‐Range Neural and Gap Junction Protein‐Mediated Cues Control Polarity During Planarian Regeneration,” Developmental Biology 339 (2010): 188–199. 20026026 10.1016/j.ydbio.2009.12.012 PMC2823934

[133] J. Beisson , “Preformed Cell Structure and Cell Heredity,” Prion 2 (2008): 1–8. 19164887 10.4161/pri.2.1.5063 PMC2634414

[134] J. Beisson and T. M. Sonneborn , “Cytoplasmic Inheritance of the Organization of the Cell Cortex in Paramecium Aurelia,” Proceedings of the National Academy of Sciences 53 (1965): 275–282. 10.1073/pnas.53.2.275 PMC219507 14294056

[135] A. B. Bubenik and R. Pavlansky , “Trophic Responses to Trauma in Growing Antlers,” Journal of Experimental Zoology 159 (1965): 289–302. 5883952 10.1002/jez.1401590302

[136] D. Lobo , M. Solano , G. A. Bubenik , and M. Levin , “A Linear‐Encoding Model Explains the Variability of the Target Morphology in Regeneration,” Journal of the Royal Society, Interface /the Royal Society 11 (2014): 20130918. 10.1098/rsif.2013.0918 PMC3899861 24402915

[137] K. J. Pienta , B. A. Robertson , D. S. Coffey , and R. S. Taichman , “The Cancer Diaspora: Metastasis beyond the Seed and Soil Hypothesis,” Clinical Cancer Research 19 (2013): 5849–5855. 24100626 10.1158/1078-0432.CCR-13-2158 PMC3835696

[138] C. A. Aktipis , A. M. Boddy , R. A. Gatenby , J. S. Brown , and C. C. Maley , “Life History Trade‐Offs in Cancer Evolution,” Nature Reviews Cancer 13 (2013): 883–892. 24213474 10.1038/nrc3606 PMC4010142

[139] M. D. Vincent , “Cancer: Beyond Speciation,” Advances in Cancer Research 112 (2011): 283–350. 21925308 10.1016/B978-0-12-387688-1.00010-7

[140] R. A. Gatenby , “Is the Genetic Paradigm of Cancer Complete?” Radiology 284 (2017): 1–3. 28628406 10.1148/radiol.2017171004

[141] K. Del Rio‐Tsonis and P. A. Tsonis , “Amphibian Tissue Regeneration—A Model for Cancer Regulation,” International Journal of Oncology 1 (1992): 161–164. 21584526 10.3892/ijo.1.2.161

[142] R. T. Prehn , “Regeneration versus Neoplastic Growth,” Carcinogenesis 18 (1997): 1439–1444. 9276613 10.1093/carcin/18.8.1439

[143] J. C. Kasemeier‐Kulesa , J. M. Teddy , L. M. Postovit , et al., “Reprogramming Multipotent Tumor Cells With the Embryonic Neural Crest Microenvironment,” Developmental Dynamics 237 (2008): 2657–2666. 18629870 10.1002/dvdy.21613 PMC2570047

[144] C. H. Waddington , “Cancer and the Theory of Organisers,” Nature 135 (1935): 606–608.

[145] H. S. Burr , “Biologic Organization and the Cancer Problem,” Yale Journal of Biology and Medicine 12 (1940): 277–282. 21433882 PMC2602176

[146] H. Rubin , “Ordered Heterogeneity and Its Decline in Cancer and Aging,” Advances in Cancer Research 98 (2007): 117–147. 17433909 10.1016/S0065-230X(06)98004-X

[147] H. Rubin , “What Keeps Cells in Tissues Behaving Normally in the Face of Myriad Mutations?” BioEssays 28 (2006): 515–524. 16615084 10.1002/bies.20403

[148] B. T. Chernet and M. Levin , “Endogenous Voltage Potentials and the Microenvironment: Bioelectric Signals That Reveal, Induce and Normalize Cancer,” Journal of Clinical & Experimental Oncology S1 (2013): S1–S2. 10.4172/2324-9110.S1-002 PMC4267524 25525610

[149] M. Lobikin , B. T. Chernet , D. Lobo , and M. Levin , “Resting Potential, Oncogene‐Induced Tumorigenesis, and Metastasis: The Bioelectric Basis of Cancer in Vivo,” Physical Biology 9 (2012): 065002. 23196890 10.1088/1478-3975/9/6/065002 PMC3528107

[150] M. Levin , “Bioelectrical Approaches to Cancer as a Problem of the Scaling of the Cellular Self,” Progress in Biophysics and Molecular Biology 165 (2021): 102–113. 33961843 10.1016/j.pbiomolbio.2021.04.007

[151] L. Cisneros , K. J. Bussey , A. J. Orr , M. Miocevic , C. H. Lineweaver , and P. Davies , “Ancient Genes Establish Stress‐Induced Mutation as a Hallmark of Cancer,” PLoS ONE 12 (2017): e0176258. 28441401 10.1371/journal.pone.0176258 PMC5404761

[152] K. J. Bussey , L. H. Cisneros , C. H. Lineweaver , and P. C. W. Davies , “Ancestral Gene Regulatory Networks Drive Cancer,” Proceedings of the National Academy of Sciences of the United States of America 114 (2017): 6160–6162. 28584134 10.1073/pnas.1706990114 PMC5474827

[153] P. C. W. Davies and C. H. Lineweaver , “Cancer Tumors as Metazoa 1.0: Tapping Genes of Ancient Ancestors,” Physical Biology 8 (2011): 015001. 21301065 10.1088/1478-3975/8/1/015001 PMC3148211

[154] M. Bizzarri and A. Cucina , “SMT and TOFT: Why and How They Are Opposite and Incompatible Paradigms,” Acta Biotheoretica 64 (2016): 221–239. 27283400 10.1007/s10441-016-9281-4

[155] C. Sonnenschein , A. M. Soto , A. Rangarajan , and P. Kulkarni , “Competing Views on Cancer,” Journal of Biosciences 39 (2014): 281–302. 24736160 10.1007/s12038-013-9403-y PMC4136489

[156] A. M. Soto and C. Sonnenschein , “Paradoxes in Carcinogenesis: There Is Light at the End of that Tunnel!” Disruptive Science and Technology 1 (2013): 154–156. 24587978 10.1089/dst.2013.0008 PMC3938290

[157] S. G. Baker , “Paradox‐Driven Cancer Research,” Disruptive Science and Technology 1 (2013): 143–148.

[158] A. M. Soto and C. Sonnenschein , “The Tissue Organization Field Theory of Cancer: A Testable Replacement for the Somatic Mutation Theory,” BioEssays 33 (2011): 332–340. 21503935 10.1002/bies.201100025 PMC3933226

[159] V. Krutovskikh and H. Yamasaki , “The Role of Gap Junctional Intercellular Communication (GJIC) Disorders in Experimental and Human Carcinogenesis,” Histology and Histopathology 12 (1997): 761–768. 9225159

[160] M. Mesnil , S. Crespin , J. L. Avanzo , and Z.‐D. ML , “Defective Gap Junctional Intercellular Communication in the Carcinogenic Process,” Biochimica Et Biophysica Acta 1719 (2005): 125–145. 16359943 10.1016/j.bbamem.2005.11.004

[161] F. Bischoff and G. Bryson , “Carcinogenesis through Solid State Surfaces,” Progress in Experimental Tumor Research 5 (1964): 85–133. 14317768 10.1159/000385997

[162] B. S. Oppenheimer , E. T. Oppenheimer , and A. P. Stout , “Sarcomas Induced in Rodents by Imbedding Various Plastic Films,” Proceedings of the Society for Experimental Biology and Medicine 79 (1952): 366–369. 14920429 10.3181/00379727-79-19380

[163] B. S. Oppenheimer , E. T. Oppenheimer , and A. P. Stout , “Sarcomas Induced in Rats by Implanting Cellophane,” Proceedings of the Society for Experimental Biology and Medicine 67 (1948): 33. 18902284 10.3181/00379727-67-16195p

[164] L. Zuniga , A. Cayo , W. Gonzalez , C. Vilos , et al., “Potassium Channels as a Target for Cancer Therapy: Current Perspectives,” OncoTargets and Therapy 15 (2022): 783–797. 35899081 10.2147/OTT.S326614 PMC9309325

[165] N. Prevarskaya , R. Skryma , and Y. Shuba , “Ion Channels in Cancer: Are Cancer Hallmarks Oncochannelopathies?” Physiological Reviews 98 (2018): 559–621. 29412049 10.1152/physrev.00044.2016

[166] S. Gentile , “hERG1 potassium Channel in Cancer Cells: A Tool to Reprogram Immortality,” European Biophysics Journal 45 (2016): 649–655. 27649700 10.1007/s00249-016-1169-3

[167] V. R. Rao , M. Perez‐Neut , S. Kaja , and S. Gentile , “Voltage‐Gated Ion Channels in Cancer Cell Proliferation,” Cancers 7 (2015): 849–875. 26010603 10.3390/cancers7020813 PMC4491688

[168] A. Litan and S. A. Langhans , “Cancer as a Channelopathy: Ion Channels and Pumps in Tumor Development and Progression,” Frontiers in cellular neuroscience 9 (2015): 86. 25852478 10.3389/fncel.2015.00086 PMC4362317

[169] V. P. Kale , S. G. Amin , and M. K. Pandey , “Targeting Ion Channels for Cancer Therapy by Repurposing the Approved Drugs,” Biochimica Et Biophysica Acta 1848 (2015): 2747–2755. 25843679 10.1016/j.bbamem.2015.03.034

[170] F. H. Mohammed , M. A. Khajah , M. Yang , W. J. Brackenbury , and Y. A. Luqmani , “Blockade of Voltage‐Gated Sodium Channels Inhibits Invasion of Endocrine‐Resistant Breast Cancer Cells,” International Journal of Oncology 48 (2016): 73–83. 26718772 10.3892/ijo.2015.3239 PMC4734602

[171] C. Fairhurst , F. Martin , I. Watt , T. Doran , M. Bland , and W. J. Brackenbury , “Sodium Channel‐Inhibiting Drugs and Cancer Survival: Protocol for a Cohort Study Using the CPRD Primary Care Database,” BMJ Open 6 (2016): e011661. 10.1136/bmjopen-2016-011661 PMC5020752 27601493

[172] S. Yildirim , S. Altun , H. Gumushan , A. Patel , and M. B. A. Djamgoz , “Voltage‐Gated Sodium Channel Activity Promotes Prostate Cancer Metastasis in Vivo,” Cancer Letters 323 (2012): 58–61. 22484465 10.1016/j.canlet.2012.03.036

[173] D. Niraula , I. El Naqa , and J. A. Tuszynski , and R. A. Gatenby , “Modeling Non‐Genetic Information Dynamics in Cells Using Reservoir Computing,” Iscience 27 (2024): 109614. 38632985 10.1016/j.isci.2024.109614 PMC11022048

[174] R. A. Gatenby and B. R. Frieden , “Cellular Information Dynamics Through Transmembrane Flow of Ions,” Scientific Reports 7 (2017): 15075. 29118414 10.1038/s41598-017-15182-2 PMC5678125

[175] B. T. Chernet and M. Levin , “Transmembrane Voltage Potential Is an Essential Cellular Parameter for the Detection and Control of Tumor Development in a Xenopus Model,” Disease Models & Mechanisms 6 (2013): 595–607. 23471912 10.1242/dmm.010835 PMC3634644

[176] D. Blackiston , D. S. Adams , J. M. Lemire , M. Lobikin , and M. Levin , “Transmembrane Potential of GlyCl‐Expressing Instructor Cells Induces a Neoplastic‐Like Conversion of Melanocytes via a Serotonergic Pathway,” Disease Models & Mechanisms 4 (2011): 67–85. 20959630 10.1242/dmm.005561 PMC3008964

[177] D. Lobo , M. Lobikin , and M. Levin , “Discovering Novel Phenotypes With Automatically Inferred Dynamic Models: A Partial Melanocyte Conversion in Xenopus,” Scientific Reports 7 (2017): 41339. 28128301 10.1038/srep41339 PMC5269672

[178] M. Lobikin , D. Lobo , D. J. Blackiston , C. J. Martyniuk , E. Tkachenko , and M. Levin , “Serotonergic Regulation of Melanocyte Conversion: A Bioelectrically Regulated Network for Stochastic All‐or‐None Hyperpigmentation,” Science Signaling 8 (2015): ra99. 26443706 10.1126/scisignal.aac6609

[179] B. T. Chernet , D. S. Adams , M. Lobikin , and M. Levin , “Use of Genetically Encoded, Light‐Gated Ion Translocators to Control Tumorigenesis,” Oncotarget 7 (2016): 19575–19588. 26988909 10.18632/oncotarget.8036 PMC4991402

[180] B. T. Chernet , C. Fields , and M. Levin , “Long‐Range Gap Junctional Signaling Controls Oncogene‐Mediated Tumorigenesis in Xenopus laevis Embryos,” Frontiers in Physiology 5 (2015): 519. 25646081 10.3389/fphys.2014.00519 PMC4298169

[181] B. T. Chernet and M. Levin , “Transmembrane Voltage Potential of Somatic Cells Controls Oncogene‐Mediated Tumorigenesis at Long‐Range,” Oncotarget 5 (2014): 3287–3306. 24830454 10.18632/oncotarget.1935 PMC4102810

[182] A. Felipe , R. Vicente , N. Villalonga , et al., “Potassium Channels: New Targets in Cancer Therapy,” Cancer Detection and Prevention 30 (2006): 375–385. 16971052 10.1016/j.cdp.2006.06.002

[183] L. Pio‐Lopez , and M. Levin , “Morphoceuticals: Perspectives for discovery of drugs targeting anatomical control mechanisms in regenerative medicine, cancer and aging,” Drug Discovery Today 28 (2023): 103585. 37059328 10.1016/j.drudis.2023.103585

[184] S. A. Newman , “Inherency of Form and Function in Animal Development and Evolution,” Frontiers in Physiology 10 (2019): 702. 31275153 10.3389/fphys.2019.00702 PMC6593199

[185] S. A. Newman , “Inherency and Homomorphy in the Evolution of Development,” Current Opinion in Genetics & Development 57 (2019): 1–8. 31302471 10.1016/j.gde.2019.05.006

[186] S. A. Newman . 2017. “Inherency,” in Evolutionary Developmental Biology: A Reference Guide, eds. L. Nuno de la Rosa and G. Müller (Cham: Springer International Publishing, 2017), 1–12.

[187] O. Gatjens‐Boniche , “The Mechanism of Plant Gall Induction by Insects: Revealing Clues, Facts, and Consequences in a Cross‐Kingdom Complex Interaction,” Revista De Biologia Tropical 67 (2019): 1359–1382.

[188] G. Gumuskaya , P. Srivastava , B. G. Cooper , et al., “Motile Living Biobots Self‐Construct from Adult Human Somatic Progenitor Seed Cells,” Advancement of Science 11, no. 4 (2024): e2303575. 10.1002/advs.202303575 PMC10811512 38032125

[189] J. Liu , R. Martinez‐Corral , A. Prindle , et al., “Coupling between Distant Biofilms and Emergence of Nutrient Time‐Sharing,” Science 356 (2017): 638–642. 28386026 10.1126/science.aah4204 PMC5645014

[190] E. Hoel and M. Levin , “Emergence of Informative Higher Scales in Biological Systems: A Computational Toolkit for Optimal Prediction and Control,” Communicative & Integrative Biology 13 (2020): 108–118. 33014263 10.1080/19420889.2020.1802914 PMC7518458

[191] B. Klein and E. Hoel . 2019. “Uncertainty and Causal Emergence in Complex Networks.” arXiv e‐prints.

[192] G. Tononi , G. M. Edelman , and O. Sporns , “Complexity and Coherency: Integrating Information in the Brain,” Trends in Cognitive Sciences 2 (1998): 474–484. 21227298 10.1016/s1364-6613(98)01259-5

[193] G. Tononi , O. Sporns , and G. M. Edelman , “A Measure for Brain Complexity: Relating Functional Segregation and Integration in the Nervous System,” Proceedings of the National Academy of Sciences 91 (1994): 5033–5037. 10.1073/pnas.91.11.5033 PMC43925 8197179

[194] Y. Fan , C. Chai , P. Li , X. Zou , J. E. Ferrell Jr , and B. Wang , “Ultrafast Distant Wound Response Is Essential for Whole‐Body Regeneration,” Cell 186 (2023): 3606–3618 e16. 10.1016/j.cell.2023.06.019 PMC10957142 37480850

[195] V. P. Pai , J. M. Lemire , Y. Chen , G. Lin , and M. Levin , “Local and Long‐Range Endogenous Resting Potential Gradients Antagonistically Regulate Apoptosis and Proliferation in the Embryonic CNS,” International Journal of Developmental Biology 59 (2015): 327–340. 26198142 10.1387/ijdb.150197ml PMC10505512

[196] C. Fields and M. Levin , “Scale‐Free Biology: Integrating Evolutionary and Developmental Thinking,” BioEssays 42 (2020): e1900228. 32537770 10.1002/bies.201900228

[197] A. Tung , M. Sperry , W. Clawson , et al., “Embryos Assist each Other's Morphogenesis: Calcium and ATP Signaling Mechanisms in Collective Resistance to Teratogens,” Nature Communications 15, no. 1 (2023): 535. 10.1038/s41467-023-44522-2 PMC10794468 38233424

[198] Z. A. Bacigalupa , M. D. Landis , and J. C. Rathmell , “Nutrient Inputs and Social Metabolic Control of T Cell Fate,” Cell Metabolism 36 (2024): 10–20. 38118440 10.1016/j.cmet.2023.12.009 PMC10872404

[199] D. J. Blackiston , K. Vien , and M. Levin , “Serotonergic Stimulation Induces Nerve Growth and Promotes Visual Learning via Posterior Eye Grafts in a Vertebrate Model of Induced Sensory Plasticity,” NPJ Regenerative Medicine 2 (2017): 8. 29302344 10.1038/s41536-017-0012-5 PMC5665622

[200] A. Tseng and M. Levin , “Cracking the Bioelectric Code: Probing Endogenous Ionic Controls of Pattern Formation,” Communicative & Integrative Biology 6 (2013): 1–8. 10.4161/cib.22595 PMC3689572 23802040

[201] J. L. Perez Velazquez , “Dynamiceuticals: The Next Stage in Personalized Medicine,” Frontiers in Neuroscience 11 (2017): 329. 28638319 10.3389/fnins.2017.00329 PMC5461286

[202] M. Etcheverry , C. Moulin‐Frier , P.‐Y. Oudeyer , and M. Levin , “AI‐Driven Automated Discovery Tools Reveal Diverse Behavioral Competencies of Biological Networks,” Elife 13 (2024): RP92683. 10.7554/eLife.92683 PMC11729405 39804159

[203] R. Rubin , “Could GLP‐1 Receptor Agonists like Semaglutide Treat Addiction, Alzheimer Disease, and Other Conditions?” Journal of American Medical Association 331 (2024): 1519–1521. 10.1001/jama.2024.1017 38639975

[204] J. Nicolau , M. I. Tamayo , P. Sanchis , et al., “Short‐Term Effects of Semaglutide among Patients With Obesity With and Without Food Addiction: An Observational Study,” Journal of Addictive Diseases 42 (2024): 1–9. 38369467 10.1080/10550887.2024.2315365

[205] M. Leslie , “Hot Weight Loss Drugs Tested against Addiction,” Science 381 (2023): 930–931. 37651529 10.1126/science.adk5720

[206] D. Moore , S. I. Walker , and M. Levin , “Cancer as a Disorder of Patterning Information: Computational and Biophysical Perspectives on the Cancer Problem,” Convergent Science Physical Oncology 3 (2017): 043001.

[207] C. Ly , A. C. Greb , L. P. Cameron , et al., “ *Psychedelics Promote Structural and Functional Neural Plastici*t*y* ,” Cell reports 23 (2018): 3170–3182. 29898390 10.1016/j.celrep.2018.05.022 PMC6082376

[208] A. Inserra , “Hypothesis: The Psychedelic Ayahuasca Heals Traumatic Memories via a Sigma 1 Receptor‐Mediated Epigenetic‐Mnemonic Process,” Frontiers in Pharmacology 9 (2018): 330. 29674970 10.3389/fphar.2018.00330 PMC5895707

[209] F. Sampaziotis , D. Muraro , O. C. Tysoe , et al., “Cholangiocyte Organoids Can Repair Bile Ducts after Transplantation in the Human Liver,” Science 371 (2021): 839–846. 33602855 10.1126/science.aaz6964 PMC7610478

[210] J. P. Sturmberg , J. M. Bennett , C. M. Martin , and M. Picard , “‘Multimorbidity’ as the Manifestation of Network Disturbances,” Journal of Evaluation in Clinical Practice 23 (2017): 199–208. 27421249 10.1111/jep.12587

[211] U. Alon , Periodic Table of Diseases (Boca Raton, FL: Chapman and Hall/CRC, 2023).

[212] C. L. Yntema , “Regeneration in Sparsely Innervated and Aneurogenic Forelimbs of Amblystoma Larvae,” Journal of Experimental Zoology 140 (1959): 101–123. 13846546 10.1002/jez.1401400106

[213] C. L. Yntema , “Blastema Formation in Sparsely Innervated and Aneurogenic Forelimbs of Amblystoma Larvae,” Journal of Experimental Zoology 142 (1959): 423–439. 13787331 10.1002/jez.1401420119

[214] S. Filoni , C. P. Velloso , S. Bernardini , and S. M. Cannata , “Acquisition of Nerve Dependence for the Formation of a Regeneration Blastema in Amputated Hindlimbs of Larval Xenopus Laevis: The Role of Limb Innervation and That of Limb Differentiation,” Journal of Experimental Zoology 273 (1995): 327–341. 8530914 10.1002/jez.1402730407

[215] P. W. Kalivas , S. L. Gourley , and M. P. Paulus , “Intrusive Thinking: Circuit and Synaptic Mechanisms of a Transdiagnostic Psychiatric Symptom,” Neuroscience and Biobehavioral Reviews 150 (2023): 105196. 37094741 10.1016/j.neubiorev.2023.105196 PMC10249786

[216] I. Prigogine , “Life and Physics,” Cell Biophysics 9 (1986): 217–224. 2436795 10.1007/BF02797383

[217] I. Prigogine and G. Nicolis , “Biological Order, Structure and Instabilities,” Quarterly Reviews of Biophysics 4 (1971): 107–148. 4257403 10.1017/s0033583500000615

[218] A. Goldbeter , “Dissipative Structures in Biological Systems: Bistability, Oscillations, Spatial Patterns and Waves,” Philosophical Transactions of the Royal Society A: Mathematical, Physical and Engineering Sciences 376 (2018): 20170376. 10.1098/rsta.2017.0376 PMC6000149 29891498

[219] V. N. Belintsev , “Dissipative Structures and the Problem of Biological Pattern Formation,” Usp Fiz Nauk+ 141 (1983): 55–101.

[220] H. M. McNamara , R. Salegame , and Z. A. Tanoury , “Bioelectrical Domain Walls in Homogeneous Tissues,” Nature Physics 16 (2020): 357–364. 33790984 10.1038/s41567-019-0765-4 PMC8008956

[221] T. Sobayo and D. J. Mogul , “Rapid Onset of a Kainate‐Induced Mirror Focus in Rat Hippocampus Is Mediated by Contralateral AMPA Receptors,” Epilepsy Research 106 (2013): 35–46. 23668947 10.1016/j.eplepsyres.2013.03.010

[222] I. Khalilov , G. L. Holmes , and Y. Ben‐Ari , “In Vitro Formation of a Secondary Epileptogenic Mirror Focus by Interhippocampal Propagation of Seizures,” Nature Neuroscience 6 (2003): 1079–1085. 14502289 10.1038/nn1125

[223] B. J. Wilder , “The Mirror Focus and Secondary Epileptogenesis,” International Review of Neurobiology 45 (2001): 435–446. 11130910 10.1016/s0074-7742(01)45022-7

[224] F. Morrell and L. deToledo‐Morrell , “From Mirror Focus to Secondary Epileptogenesis in Man: An Historical Review,” Advances in Neurology 81 (1999): 11–23. 10608998

[225] B. J. Wilder and F. Morrell , “Analysis of Single Cell Activity in the Mirror Focus of the Frog,” Electroencephalography and Clinical Neurophysiology 23 (1967): 84. 4165589

[226] F. Morrell , “Secondary Epileptogenic Lesions,” Epilepsia 1 (1960): 538–560. 14424294 10.1111/j.1528-1157.1959.tb04288.x

[227] F. Morrell , “Secondary Epileptogenesis in Man,” Archives of Neurology 42 (1985): 318–335. 3921008 10.1001/archneur.1985.04060040028009

[228] F. Schoeller , A. H. Horowitz , A. Jain , et al., “Interoceptive Technologies for Psychiatric Interventions: From Diagnosis to Clinical Applications,” Neuroscience and Biobehavioral Reviews 156 (2024): 105478. 38007168 10.1016/j.neubiorev.2023.105478

[229] A. Peters , B. S. McEwen , and K. Friston , “Uncertainty and Stress: Why It Causes Diseases and How It Is Mastered by the Brain,” Progress in Neurobiology 156 (2017): 164–188. 28576664 10.1016/j.pneurobio.2017.05.004

[230] P. R. Montague , R. J. Dolan , K. J. Friston , and P. Dayan , “Computational Psychiatry,” Trends in Cognitive Sciences 16 (2012): 72–80. 22177032 10.1016/j.tics.2011.11.018 PMC3556822

[231] R. A. Liversage , M. Crawford , and D. S. Mclaughlin , “Effects of Concomitant Denervation and Re‐Amputation through the Regenerative Forelimb Outgrowth in Xenopus laevis Froglets,” Canadian Journal of Zoology – Reviews of Canadian Zoology 64 (1986): 258–262.

[232] R. A. Liversage and C. Tsilfidis , “Denervation and Concomitant Amputation of Advanced Forelimb Regenerative Outgrowths in Adult Xenopus Laevis,” Canadian Journal of Zoology – Reviews of Canadian Zoology 73 (1995): 810–814.

[233] O. E. Schotte and E. G. Butler , “Phases in Regeneration of the Urodele Limb and Their Dependence Upon the Nervous System,” Journal of Experimental Zoology 97 (1944): 95–121.

[234] R. A. Liversage and D. S. McLaughlin , “Effects of Delayed Amputation on Denervated Forelimbs of Adult Newt,” Journal of Embryology and Experimental Morphology 75 (1983): 1–10. 6886605

[235] L. Pio‐Lopez and M. Levin , “Aging as a Loss of Morphostatic Information: A Developmental Bioelectricity Perspective,” Ageing Research Reviews 97 (2024): 102310. 38636560 10.1016/j.arr.2024.102310

[236] S. A. Lelievre , V. M. Weaver , J. A. Nickerson , et al., “Tissue Phenotype Depends on Reciprocal Interactions between the Extracellular Matrix and the Structural Organization of the Nucleus,” Proceedings of the National Academy of Sciences 95 (1998): 14711–14716. 10.1073/pnas.95.25.14711 PMC24514 9843954

[237] V. M. Weaver , O. W. Petersen , F. Wang , et al., “Reversion of the Malignant Phenotype of Human Breast Cells in Three‐Dimensional Culture and in Vivo by Integrin Blocking Antibodies,” Journal of Cell Biology 137 (1997): 231–245. 9105051 10.1083/jcb.137.1.231 PMC2139858

[238] T. D. Tlsty and P. W. Hein , “Know Thy Neighbor: Stromal Cells Can Contribute Oncogenic Signals,” Current Opinion in Genetics & Development 11 (2001): 54–59. 11163151 10.1016/s0959-437x(00)00156-8

[239] N. Grunberg , O. Levi‐Galibov , and R. Scherz‐Shouval , “The Role of HSF1 and the Chaperone Network in the Tumor Microenvironment,” Advances in Experimental Medicine and Biology 1243 (2020): 101–111. 32297214 10.1007/978-3-030-40204-4_7

[240] H. Y. Lu , E. S. Lorenc , H. Zhu , et al., “Multi‐Scale Neural Decoding and Analysis,” Journal of Neural Engineering 18 (2021): 045013. 10.1088/1741-2552/ac160f PMC8840800 34284369

[241] A. G. Huth , T. Lee , S. Nishimoto , N. Y. Bilenko , A. T. Vu , and J. L. Gallant , “Decoding the Semantic Content of Natural Movies from Human Brain Activity,” Frontiers in Systems Neuroscience 10 (2016): 81. 27781035 10.3389/fnsys.2016.00081 PMC5057448

[242] S. Nishimoto , A. T. Vu , T. Naselaris , Y. Benjamini , B. Yu , and J. L. Gallant , “Reconstructing Visual Experiences from Brain Activity Evoked by Natural Movies,” Current Biology: CB 21 (2011): 1641–1646. 21945275 10.1016/j.cub.2011.08.031 PMC3326357

[243] T. Naselaris , R. J. Prenger , K. N. Kay , M. Oliver , and J. L. Gallant , “Bayesian Reconstruction of Natural Images from Human Brain Activity,” Neuron 63 (2009): 902–915. 19778517 10.1016/j.neuron.2009.09.006 PMC5553889

[244] A. A. Mason , “Case of Congenital Ichthyosiform Erythrodermia of Brocq Treated by Hypnosis,” British Medical Journal 2 (1952): 422–423. 14944845 10.1136/bmj.2.4781.422 PMC2021155

[245] P. D. Shenefelt , “Psychological Interventions in the Management of Common Skin Conditions,” Psychology Research and Behavior Management 3 (2010): 51–63. 22110329 10.2147/prbm.s7072 PMC3218765

[246] W. T. Tsushima , “Current Psychological Treatments for Stress‐Related Skin Disorders,” Cutis: Cutaneous Medicine for the Practitioner 42 (1988): 402–404. 3058396

[247] I. O. Azuonye , “A Difficult Case: Diagnosis Made by Hallucinatory Voices,” British Medical Journal 315 (1997): 1685–1686. 9448541 10.1136/bmj.315.7123.1685 PMC2128009

[248] C. Flock , M. Grapp , R. Oldsen , H.‐C. Friederich , and T. J. Bugaj , “Therapeutic Alliance in Psycho‐Oncology: A Systematic Review,” Counselling and Psychotherapy Research 24, no. 4 (2023): 1149–1167.

[249] P. D. Shenefelt , “Hypnosis in Dermatology,” Archives of Dermatology 136 (2000): 393–399. 10724204 10.1001/archderm.136.3.393

[250] M. L. Dossett , G. L. Fricchione , and H. Benson , “A New Era for Mind–Body Medicine,” New England Journal of Medicine 382 (2020): 1390–1391. 32268025 10.1056/NEJMp1917461 PMC7486127

[251] M. P. Rogers , J. R. Peteet , and P. Reich , “Conditioned Immunosuppression?” American Journal of Psychiatry 140 (1983): 1110–1111. 10.1176/ajp.140.8.1110b 6869614

[252] M. P. Rogers , D. Dubey , and P. Reich , “The Influence of the Psyche and the Brain on Immunity and Disease Susceptibility: A Critical Review,” Psychosomatic Medicine 41 (1979): 147–164. 375277 10.1097/00006842-197903000-00008

[253] M. P. Rogers , P. Reich , T. B. Strom , and C. B. Carpenter , “Behaviorally Conditioned Immunosuppression: Replication of a Recent Study,” Psychosomatic Medicine 38 (1976): 447–451. 1005635 10.1097/00006842-197611000-00009

[254] S. Balasubramanian , D. A. Weston , M. Levin , and D. C. C. Davidian , “Electroceuticals: Emerging Applications beyond the Nervous System and Excitable Tissues,” Trends in Pharmacological Sciences 45 (2024): 391–394. 38641490 10.1016/j.tips.2024.03.001

[255] M. Kirschner , J. Gerhart , and T. Mitchison , “Molecular “Vitalism”,” Cell 100 (2000): 79–88. 10647933 10.1016/s0092-8674(00)81685-2

[256] E. F Keller . 2010. “It Is Possible to Reduce Biological Explanations to Explanations in Chemistry and/or Physics,” in Contemporary Debates in Philosophy of Biology, eds. F. J. Ayala , and R. Arp (Hoboken, NJ: Wiley‐Blackwell, 2010), 19–31.

[257] P. Nurse , “Reductionism and Explanation in Cell Biology,” Novartis Foundation Symposium 213 (1998): 93–101. discussion 2–5. 9653717 10.1002/9780470515488.ch7

[258] M. Delbruck , “A Physicist's Renewed Look at Biology: Twenty Years Later,” Science 168 (1970): 1312–1315. 5444262 10.1126/science.168.3937.1312

[259] J. Monod , Chance and Necessity: An Essay on the Natural Philosophy of Modern Biology (New York: Vintage Books, 1972).

[260] R. Rosen , Anticipatory Systems: Philosophical, Mathematical, and Methodological Foundations (Berlin: Springer, 2012).

[261] H. R. Maturana and F. J. Varela , Autopoiesis and Cognition the Realization of the Living. Boston Studies in the Philosophy of Science (Dordrecht: Springer Netherlands, 1980).

[262] F. G. Varela , H. R. Maturana , and R. Uribe , “Autopoiesis: The Organization of Living Systems, Its Characterization and a Model,” Currents in Modern Biology 5 (1974): 187–196. 4407425 10.1016/0303-2647(74)90031-8

[263] D. Noble , Dance to the Tune of Life: Biological Relativity Cambridge (United Kingdom: Cambridge University Press, 2017).

[264] M.‐W. Ho , The Rainbow and the Worm: The Physics of Organisms Singapore (River Edge, NJ: World Scientific, 1993).

[265] M.‐W. Ho and S. W. Fox , Evolutionary Processes and Metaphors Chichester (New York: Wiley, 1988).

[266] E. F. Keller . A Feeling for the Organism: The Life and Work of Barbara McClintock (Boston, MA: Northeastern University, 1984).

[267] S. Gissis and E. Jablonka , Transformations of Lamarckism: From Subtle Fluids to Molecular Biology (Cambridge, MA: MIT Press, 2011).

[268] A. Rosenberg , Darwinian Reductionism, or, How to Stop Worrying and Love Molecular Biology Chicago (Chicago, IL: University of Chicago Press, 2006).

[269] D. Noble , “The Aims of Systems Biology: Between Molecules and Organisms,” Pharmacopsychiatry 44, no. S1 (2011): S9–S14. 21544748 10.1055/s-0031-1271703

[270] B. C. Goodwin , “The Life of Form. Emergent Patterns of Morphological Transformation,” Comptes Rendus De L'academie Des Sciences Serie III 323 (2000): 15–21. 10.1016/s0764-4469(00)00107-4 10742907

[271] G. Webster and B. C. Goodwin , Form and Transformation: Generative and Relational Principles in Biology (New York: Cambridge University Press, 1996).

[272] B. C. Goodwin , How the Leopard Changed Its Spots: The Evolution of Complexity New York (New York: Charles Scribner's Sons, 1994).

[273] B. C. Goodwin , “Cognitive Biology,” Communication and Cognition 10 (1977): 87–91.

[274] R. Rosen , Anticipatory Systems: Philosophical, Mathematical, and Methodological Foundations (Oxford, England; New York: Pergamon Press, 1985).

[275] R. Rosen , “Anticipatory Systems in Retrospect and Prospect,” General Systems 24 (1979): 11–23.

[276] H. R. Maturana and F. J. Varela , Autopoiesis and Cognition: The Realization of the Living Dordrecht (Holland; Boston: D. Reidel Pub. Co, 1980).

[277] J. Bongard and M. Levin , “There's Plenty of Room Right Here: Biological Systems as Evolved, Overloaded, Multi‐Scale Machines,” Biomimetics 8 (2023): 110. 36975340 10.3390/biomimetics8010110 PMC10046700

